# Distance and similarity measures on belief and plausibility under q-rung orthopair fuzzy sets with applications

**DOI:** 10.1038/s41598-024-66555-3

**Published:** 2024-08-15

**Authors:** Rashid Hussain, Zahid Hussain, Nadia M. Sarhan, Nizomiddin Juraev, Shams Ur Rahman

**Affiliations:** 1 Department of Mathematical Sciences, Karakoram International University, Gilgit-Baltistan, Pakistan; 2 Statistics Department, Faculty of Commerce, Al-Azhar University, Cairo, Egypt; 3 Quantitative Analysis Department, College of Business Administration, King Saud University, Riyadh, Saudi Arabia; 4 Faculty of Chemical Engineering, New Uzbekistan University, Tashkent, Uzbekistan; 5 Scientific and Innovation Department, Tashkent State Pedagogical University, Tashkent, Uzbekistan

**Keywords:** q-ROFSs, Belief and plausibility, Pattern recognition, Clustering, MCDM, OBP-GRA, Diseases, Energy science and technology, Mathematics and computing

## Abstract

Belief and plausibility functions based on evidence theory (ET) have been widely used in managing uncertainty. Various generalizations of ET to fuzzy sets (FSs) have been reported in the literature, but no generalization of ET to q-rung orthopair fuzzy sets (q-ROFSs) has been made yet. Therefore, this paper proposes a novel, simple, and intuitive approach to distance and similarity measures for q-ROFSs based on belief and plausibility functions within the framework of ET. This research addresses a significant research gap by introducing a comprehensive framework for handling uncertainty in q-ROFSs using ET. Furthermore, it acknowledges the limitations inherent in the current state of research, notably the absence of generalizations of ET to q-ROFSs and the challenges in extending belief and plausibility measures to certain aggregation operators and other generalizations including Hesitant fuzzy sets, Bipolar fuzzy sets, Fuzzy soft sets etc. Our contribution lies in the proposal of a novel approach to distance and similarity measures for q-ROFSs under ET, utilizing Orthopairian belief and plausibility intervals (OBPIs). We establish new similarity measures within the generalized ET framework and demonstrate the reasonability of our method through useful numerical examples. Additionally, we construct Orthopairian belief and plausibility GRA (OBP-GRA) for managing daily life complex issues, particularly in multicriteria decision-making scenarios. Numerical simulations and results confirm the usability and practical applicability of our proposed method in the framework of ET.

## Introduction

There exist a lot of uncertain situations which are random, fuzzy, vague, ambiguous, unpredictable and imprecise. To handle such uncertain situations close to reality, Lotfi A. Zadeh^1^ proposed a theory named as fuzzy set (FS) in 1965, which is an extension of crisp sets. The term “fuzziness” describes the uncertainty in elements of a set. Zadeh^1^ was the first to propose FSs as an extended form of ordinary sets. In fuzzy set, we work on membership degree, and the non-membership degree is taken as (1-membership). However, in real-life scenarios, there are many situations where non-membership degree does not equal to (1-membership), so there is a need of hesitancy degree. To address this gap Atanassov^2,3^ was first to generalize the FSs to IFSs in 1986, with the consideration of membership degree $\mu$, non-membership degree $\nu$ and degree of hesitancy $\pi$ with the constraint condition $\mu + \nu + \pi = 1$ or $0 \le \mu + \nu \le 1$. The world didn’t stop with IFSs and researchers have modified the drawbacks in IFSs and later on, in 2013, Yager^4,5^ gave new concept which extends IFS to another concept, called PFSs which performs better than IFSs. The characterization of PFSs based on degrees of membership, non-membership and hesitancy, respectively with the condition that the sum of squares of all these three terms is equal to one^4–10^. In some sets, the IFSs can’t fulfill the required condition $0 \le \mu + \nu \le 1$, for example consider $A = (0.5,\,\,0.6)$, then $0 \le 0.6 + 0.5\not \le 1 = 1.1$. Yager^4,5^ suggested the notion of Pythagorean fuzzy set (PFS) which handle the complex issues and fulfill the condition $0 \le \mu^{2} + \nu^{2} \le 1$. For instance, let $A = (0.5,\,\,0.6)$, then $0 \le 0.6^{2} + 0.5^{2} \le 1 = 0.61$. A PFS can be termed mathematically as: let $\lambda$ be any PFS in G then $\lambda = \left\langle {a,\mu_{\lambda }^{2} \left( a \right),\nu^{2}_{\lambda } \left( a \right),\pi^{2}_{\lambda } \left( a \right):a \in G} \right\rangle$ and satisfies $0 \le \mu^{2} + \nu^{2} \le 1$ where degree of uncertainty is given by $\pi = \sqrt {1 - \mu^{2} - \nu^{2} }$. Furthermore, there are still some cases where PFSs are unable to handle certain situations i.e. $0 \le 0.8^{2} + 0.7^{2} = 1.13\not \le 1$. There was a need of further extension of PFSs. Therefore, in 2017, Yager^11^ initialized another candid extension by taking the qth power of membership and non-membership degrees, which he named as q-rung orthopair fuzzy sets. The characterization of q-ROFSs is same as PFS but they differ by constraint condition that is $0 \le \mu^{q} + \nu^{q} \le 1.$ The uncertainty degree of q-ROFS will be $\pi = \sqrt[q]{{1 - \mu^{q} - \nu^{q} }}$. In other words, q-ROFSs are considered as the generalized forms of IFSs and PFSs. Reference^12^ gave q-rung orthopair fuzzy weighted averaging (q-ROFWA) operators and q-rung orthopair fuzzy weighted geometric (q-ROFWG) operators.

### Literature review

Distance is being used as a tool to measure difference between two objects. First distance measure is proposed by Szmidt and Kacprzyk^13^. Szmidt and Kacprzyk^13^ proposed distance by using hesitance index. Wang and Xin^14^ had criticized on the studies of Szmidt and Kacprzyk^13^ that they were not much effective. Hamming distance^15^ and Euclidean distance^16^, and after that the Hausdorff distance was proposed by Grzegorzewski^15^ which was revised by Xu and Chen^16^. Yang and Hussain^17^ and Ali et al.^18^ also worked on distance and similarity measures of HFSs. Hussain et al.^19^ worked on distance measures on q-ROFSs. Many researchers worked on distance and similarities based on q-ROFSs such as^11,12^. Jawad^20^ proposed normed-based distance measures of q-ROFSs with applications. Ejegwa^21^ proposed new q-rung orthopair fuzzy distance-similarity operators with applications. Arora et al.^22^ suggested similarity measures for q-ROFSs and applications to decision making. Ziyue & Xiao^23^ proposed generalized Hellinger distance for multisource information fusion with applications. Wang et al.^24^ proposed novel distance measures of q-ROFSs with their applications.

Belief and plausibility is another emerging field to manage uncertainty based upon ET^25–27^ and they have been widely used in many areas. This framework improves comprehension, reasoning, and decision-making across various topics, ultimately resulting in more robust and well-informed judgments. It improves the understanding and assessment of unclear information. Dealing with uncertainty is crucial in several fields, including risk analysis, artificial intelligence, and decision-making. Belief and plausibility offer a systematic framework for reasoning and making well-informed decisions in the face of contradictory or inadequate data. Researchers incorporate subjective evaluations and evaluate the viability of alternative hypotheses in an effort to reflect the inherent uncertainty in real-world scenarios. Shafer’s theory^26^ infrastructure aims to generalize probability theory, which is challenging when addressing complex issues. Dempster^25,26^ initialize the theory of lower and upper probability. Soon after Shafer^26^ introduced belief and plausibility measures on usual subsets based upon Dempster theory. However, FSs and their extensions have been shown to be very practical and sensible tools for handling various forms of uncertainty that closely resemble real-world situations. Dymova and Sevastjanov^28,29^ interpreted IFS to ET. Hwang and Yang^30^ also proposed some belief and plausibility functions which were based on the work of Dymova and Sevastjanov^28,29^. Khalaj and Khalaj^31–33^ proposed similarity measures based on belief functions. Yang and Hussain^34^ proposed theory about belief and plausible measures on IFS by constructing belief and plausible TOPSIS. Hussain et al.^35^ interpreted belief and plausible measure of PFSs with application to BPI-VIKOR. Till now, no one has considered q-ROFSs in the context of belief and plausible functions. Therefore, we dedicate this study to investigating q-ROFSs in the frame work of belief and plausible functions, particularly focusing on their application in addressing complex problems of MCCDM. We suggest normalized belief and plausible functions on q-ROFSs which enable us to construct the OBPIs. This construction allows us to create new similarity measures between belief and plausible functions by defining Hausdorff metric on OBPIs. These similarity measures^36^ are then applied in recognition of different patterns and in MCCDM by using OBP-GRA.

Multicriteria decision making (MCDM) is a systematic approach to decision making, taking into account multiple factors and criteria to compare alternatives. By considering multiple perspectives, MCDM enables decision makers to conduct a comprehensive analysis of available options, identifying the most suitable choice. This approach is particularly valuable in situations where a single optimal solution doesn’t exist. Many researchers worked on MCDM^37,38^ and applied in different fields^33,39–44^. Jana and Hezam^45^ proposed multi-attribute group decision making method for sponge iron factory location selection problem. Hagag et al.^46^ used MCDM approach for machine selection. Some other researchers worked on MCDM such as^24,47–50^.

During the Covid-19 pandemic, vaccine selection became a complex issue. Although, numerous vaccines were available but selecting the most efficient one was challenging. As far as we know, no one has suggested a methodology to select best vaccines using q-ROFSs in the frame work of belief and plausible functions. Therefore, this research addresses the selection of the most efficient vaccine to combat the pandemic.

### Research gap and limitations of existing literature

In the existing literature, no one has considered distance and similarity measures on belief and plausible functions under the context of q-ROFSs along with their applications. Also, the existing methods often fall short in effectively managing and analyzing multiple criteria under uncertainty. Additionally, no generalization of ET to q-ROFSs is reported so far.

### Motivation

The motivation behind this research work is to fill crucial gaps in existing methodologies for handling uncertainty and imprecision in complex systems. Specifically, to develop novel distance and similarity measures for belief and plausible q-ROFS based on OBPIs. Traditional approaches in pattern recognition and clustering within the framework of Evidence Theory do not adequately address the complexities and uncertainties present in real-world data. By introducing these novel measures, we aim to significantly enhance the accuracy and reliability of these applications. Furthermore, our pioneering work in constructing OBP-GRA using the proposed distance measure targets the challenges in multicriteria decision-making processes. Existing methods often fall short in effectively managing and analyzing multiple criteria under uncertainty. Our method gives decision-makers a strong and complete tool that makes use of fuzzy logic's advantages to manage uncertainty and imprecision more effectively, and develop more effective tools for multicriteria decision-making where traditional methods have proven insufficient.

### Our contribution

Our main contributions in this paper are as follow:

First, we develop novel distance and similarity measures for belief and plausible q-rung Orthopair fuzzy sets based on OBPIs which provide a more nuanced, appropriate and alternative way to measure fuzzy situations within q-ROFSs. Secondly, we apply these proposed similarity measures in pattern recognition and clustering, which is a pioneering effort that demonstrates the practical utility and effectiveness of our proposed measures in real-world applications. Finally, we construct OBP-GRA using the proposed distance measure to enhance the multi-criteria complex decision-making process, which offers a robust tool for decision-makers to evaluate and compare complex options more effectively.

### Organization of paper

The remaining article is structured as follows:

- Section "Preliminaries" consists of fundamental concepts and definitions of q-ROFSs and ET.
- Section "Novel belief and plausible measures of q-ROFSs" includes the methodology for defining belief and plausible functions in terms of q-ROFSs along with some properties.
- Section "Distance measure of q-ROFS for belief and plausibility interval" is dedicated to creating OBPIs of q-ROFSs based on the suggested belief and plausible functions. We then utilize the concept of Hausdorff distance with OBPIs to construct novel distance and similarity measures for two q-ROFSs. Furthermore, some numerical examples are given in this section to demonstrate the reasonability and usefulness of our suggested measures.
- Section "Similarity measures of q-ROFSs for belief and plausibility intervals" includes the proposed similarity measures with their applications in pattern recognition and clustering.
- Section "Construction of Orthopairian belief and plausible GRA" describes the utilization of the GRA method to create OBP-GRA for managing daily life health related complex problem involving MCCDM.
- Section "Conclusions" includes our conclusion.

## Preliminaries

We briefly describe ET and review fundamental definitions of IFSs, PFSs and q-ROFSs respectively. Then, we establish the connection between q-ROFSs and belief and plausibility functions. In a q-ROFS, the belief function is represented as membership function, and the plausibility function is represented as a non-membership function.

### Dempster-Shafer’s evidence theory

The upper and lower probabilities were first coined by Dempster^25^ with the help of multi-valued mappings. A decade later, Shafer^26^ proposed the concept of belief and plausible measures of usual subsets based upon Dempster’s probabilities. Let $\left( {\mho ,\tilde{M},P} \right)$ be a probabilistic space represented by $\tilde{\delta }$ along $\mho$ to $\tilde{\Theta }$. This will give a subset $\left\{ {\tilde{\delta }\left( \kappa \right) \subset \tilde{\Theta },\,\forall \,\,\kappa \in \mho } \right\}$ and $\tilde{\delta }$ be a set-valued function from $\mho$ to $2^{{\tilde{\Theta }}}$ of $\tilde{\Theta }$. Let $\tilde{M}$ be a subset of $\tilde{\Theta }$, then $\tilde{M}_{ * } = \left( {\kappa \in \mho :\tilde{\delta }\left( \kappa \right) \subset \tilde{M},\tilde{\delta }\left( \kappa \right) \ne \phi } \right)$, $\tilde{M}^{ * } = \left( {\kappa \in \mho :\tilde{\delta }\left( \kappa \right) \cap \tilde{M},\tilde{\delta }\left( \kappa \right) \ne \phi } \right)$. Mostly, $\tilde{\Theta }_{ * } = \tilde{\Theta }^{ * } = \mho$. Suppose $K$ be any class from $\tilde{M}$ of $\tilde{\Theta }$, and $\left\{ {\tilde{M}_{ * } ,\,\tilde{M}^{ * } } \right\} \in T$, then the probabilities $P_{ * }$ and $P^{*}$ of $\tilde{M}$ are $P_{ * } \left( {\tilde{M}} \right) = \frac{{P\left( {\tilde{M}^{ * } } \right)}}{{P\left( {\tilde{\Theta }^{ * } } \right)}}$ and $P^{ * } \left( {\tilde{M}} \right) = \frac{{P\left( {\tilde{M}^{ * } } \right)}}{{P\left( {\tilde{\Theta }^{ * } } \right)}}$ respectively if $P\left( {\tilde{\Theta }^{ * } } \right) \ne 0$ and $P_{ * } \left( {\tilde{M}} \right) + P^{ * } \left( {\tilde{M}^{c} } \right) \le 1$, $P_{ * } \left( {\tilde{M}} \right) + P^{ * } \left( {\tilde{M}^{c} } \right) \ge 1$, and $P_{ * } \left( {\tilde{M}} \right) \le P^{ * } \left( {\tilde{M}} \right)$. Let $\tilde{\delta }$ be a single-valued function, then $\tilde{\delta }$ be considered as a random variable with $P_{ * } \left( {\tilde{M}} \right) = P^{ * } \left( {\tilde{M}} \right)$. Contrarily, if $\tilde{\delta }$ be a multi-valued mapping, then $\kappa$ must have multiple mappings.

Moreover, let $\tilde{\Theta }$ be a finite set, then Shafer^26^ mapped the power set to unit interval as $m:2^{{\tilde{\Theta }}} \to [0,1]$[0, 1], with restriction of $\left( i \right)\;\;m\left( \phi \right) = 0$ and $\left( {ii} \right)\;\,\,\sum {_{{M_{i} \in 2^{\Theta } }} } m\left( {\tilde{M}_{i} } \right) = 1$ respectively. Here, $m$ is called basic probability assignment (BPA), which allocates same weights to all subsets of $\tilde{M} \subseteq \tilde{\Theta }$ from the existing evidences and $\tilde{M}$ in $2^{{\tilde{\Theta }}}$ when $m(\tilde{M}) \ne 0$ is known as focal element. Belief and plausibility measures are functions $\left\{ {Bl,Pl} \right\}:2^{{\tilde{\Theta }}} \to \left[ {0,1} \right]$ written as $Pl(\tilde{M}) = 1 - Bl(\tilde{M}^{c} )$ while $Pl(\tilde{M}) = \sum {_{N \cap M \ne \phi } } m\left( {\tilde{N}} \right) = P^{ * } \left( {\tilde{M}} \right)$. Here, $Bl(\tilde{M}) = 1 - Pl(\tilde{M}^{c} )$ and $Pl(\tilde{M}) = 1 - Bl(\tilde{M}^{c} )$.

#### Definition 1

References^2,3^ An IFS $M$ in the universal set $\tilde{Z}$ will be expressed as:

$$ M = \left\{ {\left( {z,\,\mu_{M} \left( z \right),v_{M} \left( z \right)} \right)} \right\},\,\,\,\,\forall \,z \in \,\tilde{Z} $$

such that $0 \le \mu_{M} \left( z \right) + v_{M} \left( z \right) \le 1$ where the function $\mu_{M} :\tilde{Z} \to \left[ {0,1} \right]\,$ is membership degree and $v_{M} :\tilde{Z} \to \left[ {0,1} \right]\,$ is called non-membership degree respectively.

#### Definition 2

References^4,5^ A PFS $M$ in the universe $\tilde{Z}$ is written as:

$M = \left\{ {\left\langle {z,\mu_{M}^{2} \left( z \right),v_{M}^{2} \left( z \right)} \right\rangle \,\,\,\forall \,\,z \in \tilde{Z}} \right\},$ such that $\,0 \le \mu_{{_{M} }}^{2} \left( z \right) + v_{{_{M} }}^{2} \left( z \right) \le 1$, where $\mu_{M} \left( z \right):\tilde{Z} \to \left[ {0,1} \right]$ is membership degree and $v_{M} \left( z \right):\tilde{Z} \to \left[ {0,1} \right]\,$ is non-membership degree respectively.

#### Definition 3

References^1,25^ A q- ROFS $M$ in $\tilde{Z}$ will be written as: $M = \left\{ {\left\langle {z,\mu_{M}^{q} \left( z \right),v_{M}^{q} \left( z \right)} \right\rangle \,\,\,\forall \,\,z \in \tilde{Z}} \right\},\,$ with condition $0 \le \mu_{{_{M} }}^{q} \left( z \right) + \nu_{{_{M} }}^{q} \left( z \right) \le 1$, where $\mu_{M}^{q} \left( z \right):\tilde{Z} \to \left[ {0,1} \right]$ represents the membership degree of $z \in M$, and $\nu_{M}^{q} \left( z \right):\tilde{Z} \to \left[ {0,1} \right]$ represents the non-membership degree of $z \in M$, respectively.

## Novel belief and plausible measures of q-ROFSs

First of all, q-ROFSs are interpreted under the contexts of ET and after the interpretation, the novel belief and plausibility functions on q-ROFSs are proposed. We use our proposed $Bl\left( z \right)$ and $Pl\left( z \right)$ functions to construct various similarities by using Hausdorff metric. In the framework of ET, whenever the set $M$ is taken with complement $M^{c} ,$ then the degree of belief will be such that $Bl\left( M \right) + Bl\left( {M^{c} } \right) \le 1$. Therefore, due to this reason, there is a difference between ET theory and probability theory. Under the contexts of ET, the level of commitment to a proposition's complement and its degree of belief may not be the same. For total ignorance, $Bl\left( M \right) = Bl\left( {M^{c} } \right) = 0$, but from probabilistic view point $Pro\left( M \right) = Pro\left( {M^{c} } \right) = 0.5$. So the belief function $Bl\left( M \right)$ unable to explain the belief committed to $M$ as it doesn’t explain doubt committed to $M$ that is the belief committed to $M^{c} .$ Hence, $Dou\left( M \right) = Bl\left( {M^{c} } \right)$ is defined for the sake to completely describe the belief committed to $M$. Salicone^51–53^ also discussed the doubt function.

### Linkage between evidence theory and q-ROFSs

On a close observation, it is noticed that there is a linkage between ET and q-ROFS and they are closely relate with each other. As $\mu \left( z \right)$ in q-ROFS is similar to $Bl\left( z \right)$ in ET, so we can easily relate them and can be written as $Bl_{M} \left( z \right) = \mu_{M} \left( z \right)$, which reflects that both degrees of membership and belief have a linkage and are interconnected by showing the same demonstration about certain happenings. In this scenario, $\mu_{M} \left( z \right)$ may be considered as the degree of belief of an event $z \in M$, shown by $Bl_{M} \left( z \right)$. The degree of non-membership that is one’s personal belief that the event $z \in M^{c}$ does not occur is denoted by $Bl_{{M^{c} }} \left( z \right)$. Since, in q-ROFSs, membership and non-membership degree is expressed as $\left\langle {\mu \left( z \right),\nu \left( z \right)} \right\rangle$ and its complement is denoted by $\left\langle {\nu \left( z \right),\mu \left( z \right)} \right\rangle$. The complement of an event $M$ in term of doubt’s degree is $Bl_{{M^{c} }} \left( z \right) = \nu_{M} \left( z \right)$. i.e. it may be considered as the degree of doubt that $M$ does not happen. Indeed, the $Bl_{M} \left( z \right)$ and $Bl_{{M^{c} }} \left( z \right)$ are personal judgments. So to completely describe the event $M,$ both belief function $Bl_{M} \left( z \right)$ and doubt function $Dou_{M} \left( z \right) = Bl_{{M^{c} }} \left( z \right)$ should be better given. The doubt function is rarely used. In general, a complete description of event $M$ is given by belief function $Bl_{M} \left( z \right)$ and plausibility function $Pl_{M} \left( z \right)$, and written as:

$$Pl_{M} \left( z \right) = 1 - Bl_{{M^{c} }} \left( z \right)$$$$= 1 - Dou_{M} \left( z \right)$$$$= 1 - \nu_{M} \left( z \right) = \mu_{M} \left( z \right) + \pi_{M} \left( z \right).$$ Since $$\left( {\mu \left( z \right),\nu \left( z \right),\pi \left( z \right)} \right)$$ of q-ROFSs have the condition $$\mu \left( z \right) + \nu \left( z \right) + \pi \left( z \right) = 1$$, so it properly shows basic assignment function (BAF) under the context of ET. Therefore, we express ET in terms of $$\mu \left( z \right)$$ and $$\nu \left( z \right)$$ of q-ROFSs to demonstrate a newer definition of $$Bl\left( z \right)$$ and $$Pl\left( z \right)$$ function in a very simple and intuitive way.

#### Definition 4

Suppose $\tilde{Z}$ be a universal set and $M$ be a q-ROFS in $\tilde{Z}$, then the belief function $Bl_{M} \left( z \right)$ will be,

1 $$ Bl_{M} \left( z \right) = \mu_{M}^{q} \left( z \right)\;\;\forall \;\;z \in \tilde{Z} $$

#### Definition 5

Suppose $\tilde{Z}$ is a universal set and $M$ be a q-ROFS in $\tilde{Z}$, then the plausibility function $Pl_{M} \left( z \right)$ will be,

2 $$ Pl_{M} \left( z \right) = 1 - \nu_{M}^{q} \left( z \right)\,\,\,\,\forall \,\,\,\,\,z \in \tilde{Z} $$

#### Definition 6

Let $\tilde{Z} = \left\{ {z_{1} ,z_{2} ,...,z_{n} } \right\}\,$ is a finite universes of discourse and $M$ be a q-ROFS in $\tilde{Z}$, then the belief measure $Bl$ on $M$ with the weight $\omega_{i}$ taking $\sum\limits_{i = 1}^{n} {\omega_{i} } = 1$ will be,

3 $$ Bl\left( M \right) = \sum\limits_{i = 1}^{n} {\omega_{i} .Bl_{M} \left( {z_{i} } \right)} $$

#### Definition 7

Let $\tilde{Z} = \left\{ {z_{1} ,z_{2} ,...,z_{n} } \right\}\,$ be a finite universes of discourse and $M$ be q-ROFS in $\tilde{Z}$, then the plausibility measure $Pl$ on $M$ with the weight $\omega_{i}$ taking $\sum\limits_{i = 1}^{n} {\omega_{i} } = 1$ will be,

4 $$ Pl\left( M \right) = \sum\limits_{i = 1}^{n} {\omega_{i} Pl_{M} \left( {z_{i} } \right)} \, $$

#### Definition 8

Let $Bl\left( z \right)$ and $Pl\left( z \right)$ be the belief and plausibility functions which satisfy the following properties:

$$ \left( {P_{1} } \right):0 \le Bl\left( M \right) \le Pl\left( M \right) \le 1\,; $$

$$ \left( {P_{2} } \right):\,\,\,If\,M_{1} \subseteq M_{2} \to Bl\left( {M_{1} } \right) \le Bl\left( {M_{2} } \right)\,\,{\text{and}}\,\,Pl\left( {M_{1} } \right) \le Pl\left( {M_{2} } \right)\,; $$

$$ \left( {P_{3} } \right):Bl\left( M \right) = 1 - Pl\left( {M^{c} } \right)\,\,{\text{and}}\,Pl\left( M \right) = 1 - Bl\left( {M^{c} } \right)\,; $$

$$ \left( {P_{4} } \right):Bl\left( M \right) + Bl\left( {M^{c} } \right) \le 1\,\,\,{\text{and}}\,\,\,Pl\left( M \right) + Pl\left( {M^{c} } \right) \ge 1\,. $$

#### Theorem 1

Let $\tilde{Z}$ be universal set and $M$ is subset of $\tilde{Z}$. Then, the proposed belief and plausible measures of q-ROFSs fulfill all the properties of Definition (8).

#### Property 1

$$0 \le Bl\left( M \right) \le Pl\left( M \right) \le 1\,$$.

#### Proof

For any q-ROFS it is known that $$0 \le \mu_{{_{M} }}^{q} \left( {z_{i} } \right) + \nu_{M}^{q} \left( {z_{i} } \right) + \pi_{{_{M} }}^{q} \left( {z_{i} } \right) \le 1,\forall \,z_{i} \in \tilde{Z},$$ $$\,0 \le \mu_{M}^{q} \left( {z_{i} } \right) \le 1 - \nu_{M}^{q} \left( {z_{i} } \right) \le 1$$, $$0 \le Bl_{M} \left( {z_{i} } \right) \le Pl_{M} \left( {z_{i} } \right) \le 1,\,\,$$$$\,0 \le \sum\limits_{i = 1}^{n} {\omega_{i} } Bl_{M} \left( {z_{i} } \right) \le \sum\limits_{i = 1}^{n} {\omega_{i} } Pl_{M} \left( {z_{i} } \right) \le 1$$. $$0 \le Bl\left( M \right) \le Pl\left( M \right) \le 1$$. Thus, property 1 is satisfied.

#### Property 2

$$If\,M_{1} \subseteq M_{2} \to Bl\left( {M_{1} } \right) \le Bl\left( {M_{2} } \right)\,\,{\text{and}}\,\,Pl\left( {M_{1} } \right) \le Pl\left( {M_{2} } \right)\,$$.

#### Proof

If we have two q-ROFS $M_{1}$ and $M_{2}$ which follow the condition that $M_{1} \subseteq M_{2}$ then $\mu_{{M_{1} }}^{q} \left( {z_{i} } \right) \le \mu_{{M_{2} }}^{q} \left( {z_{i} } \right),$ $\sum\limits_{i = 1}^{n} {\omega_{i} } \mu_{{M_{1} }}^{q} \left( {z_{i} } \right) \le \sum\limits_{i = 1}^{n} {\omega_{i} } \mu_{{M_{2} }}^{q} \left( {z_{i} } \right),$ $\,Bl\left( {M_{1} } \right) \le Bl\left( {M_{2} } \right)$ and similarly we can prove $Pl\left( {M_{1} } \right) \le Pl\left( {M_{2} } \right)$ by using condition $M_{1} \subseteq M_{2}$. So, property 2 is satisfied.

#### Property 3

$$Bl\left( M \right) = 1 - Pl\left( {M^{c} } \right)\,\,{\text{and}}\,Pl\left( M \right) = 1 - Bl\left( {M^{c} } \right)\,$$.

#### Proof

By previous knowledge, we can say that, $Bl\left( M \right)\, = \sum\limits_{i = 1}^{n} {\omega_{i} } \mu_{M}^{q} \left( {z_{i} } \right) = 1 - \sum\limits_{i = 1}^{n} {\omega_{i} + } \sum\limits_{i = 1}^{n} {\omega_{i} } \mu_{M}^{q} \left( {z_{i} } \right) = 1 - \sum\limits_{i = 1}^{n} {\omega_{i} \left( {1 - \mu_{M}^{q} \left( {z_{i} } \right)} \right)} = 1 - Pl\left( {M^{c} } \right)$ and similarly we can do it for the plausibility that is $Pl\left( M \right) = 1 - Bl\left( {M^{c} } \right)$. Thus, property 3 is satisfied.

#### Property 4

$$Bl\left( M \right) + Bl\left( {M^{c} } \right) \le 1\,\,\,{\text{and}}\,\,\,Pl\left( M \right) + Pl\left( {M^{c} } \right) \ge 1\,$$.

#### Proof

By definition of both membership as well as non-membership property $0 \le \mu_{M}^{q} \left( {z_{i} } \right)$ and $\,0 \le \nu_{M}^{q} \left( {z_{i} } \right) \le 1$. We can show it in the form of belief and plausibility measures that $0 \le Bl_{M} \left( {z_{i} } \right) \le 1$ and $0 \le Pl_{M} \left( {z_{i} } \right) \le 1,\,$ $Bl_{M} \left( {z_{i} } \right) \le Pl_{M} \left( {z_{i} } \right)$,$Bl_{M} \left( {z_{i} } \right) \le 1 - Bl\left( {M^{c} } \right)\,,\,\,$ since $Pl_{M} \left( {z_{i} } \right) = 1 - Bl\left( {M^{c} } \right)$, $Bl_{M} \left( {z_{i} } \right) + Bl\left( {M^{c} } \right) \le 1$ and similarly for plausibility measure we can prove it as $Bl_{M} \left( {z_{i} } \right) \le Pl_{M} \left( {z_{i} } \right),$ $1 - Pl\left( {M^{c} } \right)\, \le Pl_{M} \left( {z_{i} } \right),\,$ by combining these, we have $1 \le Pl\left( M \right) + Pl\left( {M^{c} } \right)$. Hence, all the properties are satisfied. □

#### Example 1

Consider $\tilde{Z} = \left\{ {z_{1} ,z_{2} ,z_{3} } \right\}\,$ be finite universes of discourse. Let $M$ be any q-ROFS in $\tilde{Z}$ with $M = \left\{ {\left\langle {z_{1} ,0.6,0.9} \right\rangle ,\left\langle {z_{2} ,0.7,0.8} \right\rangle ,\left\langle {z_{3} ,0.7,0.7} \right\rangle } \right\}.$ Assume that weights are assigned as $\omega_{1} = 0.6$, $\omega_{2} = 0.25$ and $\omega_{3} = 0.15$. Then from Eq. (1), the belief function will be $Bl_{M} \left( {z_{1} } \right) = 0.216,\,\,\,$ $Bl_{M} \left( {z_{2} } \right) = 0.343,\,$ and $Bl_{M} \left( {z_{3} } \right) = 0.343$ respectively. Likewise, the plausibility function will be $Pl_{M} \left( {z_{1} } \right) = 0.271,\,\,Pl_{M} \left( {z_{2} } \right) = 0.488,\,\,Pl_{M} \left( {z_{3} } \right) = 0.657$. Hence, the belief and plausible measures from belief and plausibility function of Eqs. (3) and (4) will be:

$$ Bl\left( M \right)\, = \sum\limits_{i = 1}^{n} {\omega_{i} } \mu_{M}^{q} \left( {z_{i} } \right) = \left( {0.6} \right)\left( {0.216} \right) + \left( {0.25} \right)\left( {0.343} \right) + \left( {0.15} \right)\left( {0.343} \right) = 0.2668 $$

Similarly plausibility function will be,

$$ Pl\left( M \right)\, = \sum\limits_{i = 1}^{n} {\omega_{i} } (1 - v_{M}^{q} (z)) = \left( {0.6} \right)\left( {0.271} \right) + \left( {0.25} \right)\left( {0.488} \right) + \left( {0.15} \right)\left( {0.657} \right) = 0.3832 $$

**Figure 1:**
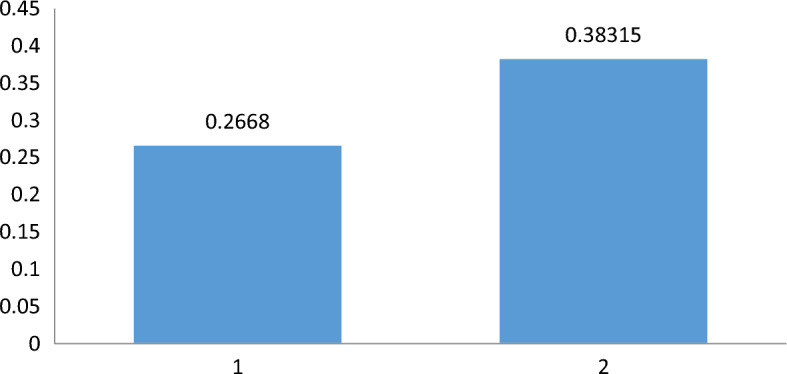
Graphical comparison of belief and plausibility measures.

The comparison is clearly shown in Fig. 1.

Therefore, the above results fulfills the condition $Bl\left( M \right)\, \le Pl\left( M \right)$.

## Distance measure of q-ROFS for belief and plausibility interval

Here, orthoparian belief and plausibility interval $OBPI_{M}$ of q-ROFSs are constructed using (1), (2). Hausdorff distance is then used to find the distance of orthoparian belief and plausibility intervals $OBPI_{M}$ and $OBPI_{N}$. Both the forward Hausdorff and backward Hausdorff distances are

5 $$ \begin{gathered} \overset{\lower0.5em\hbox{$\smash{\scriptscriptstyle\frown}$}}{H} \left( {M,N} \right) = \max_{{z_{i} \in M}} \left\{ {\min_{{z_{j} \in N}} \left( {\left\| {z_{i} - z_{j} } \right\|} \right)} \right\}, \hfill \\ \overset{\lower0.5em\hbox{$\smash{\scriptscriptstyle\frown}$}}{H} \left( {M,N} \right) = \max_{{z_{j} \in N}} \left\{ {\min_{{z_{i} \in M}} \left( {\left\| {z_{i} - z_{j} } \right\|} \right)} \right\}. \hfill \\ \end{gathered} $$

Hausdorff metric space is oriented and asymmetric, which means the forward distance $h\left( {M,N} \right)$ does not necessarily same as the backward distance $h\left( {N,M} \right)$. Hausdorff metric is defined as:

6 $$ \overset{\lower0.5em\hbox{$\smash{\scriptscriptstyle\frown}$}}{H} \left( {M,N} \right) = \max \left\{ {\overset{\lower0.5em\hbox{$\smash{\scriptscriptstyle\frown}$}}{h} \left( {M,N} \right),\overset{\lower0.5em\hbox{$\smash{\scriptscriptstyle\frown}$}}{h} \left( {N,M} \right)} \right\}.\, $$

For any two intervals $M = \left[ {m_{1} ,m_{2} } \right]\,$ and $N = \left[ {n_{1} ,n_{2} } \right]$ the Hausdorff distance will be,

7 $$ \overset{\lower0.5em\hbox{$\smash{\scriptscriptstyle\frown}$}}{H} \left( {M,N} \right) = max\left\{ {\left| {m_{1} - m_{2} } \right|,\left| {n_{1} - n_{2} } \right|} \right\}. $$

Consider $M$ and $N$ be two q-ROFSs in the universes of discourse $\tilde{Z} = \left\{ {z_{1} ,z_{2} ,...,z_{n} } \right\}\,$ for each $z_{i} \in \tilde{Z}$, we define orthoparian belief-plausibility intervals of $z_{i}$ in q-ROFS $M$ and $N$ for $i = 1,2,...,n$ as:

$$ OBPI_{M} \left( {z_{i} } \right) = \left[ {Bl_{M} \left( {z_{i} } \right),Pl_{M} \left( {z_{i} } \right)} \right] = \left[ {\mu_{M}^{q} \left( {z_{i} } \right),1 - \nu_{M}^{q} \left( {z_{i} } \right)} \right], $$

$$ OBPI_{N} \left( {z_{i} } \right) = \left[ {Bl_{N} \left( {z_{i} } \right),Pl_{N} \left( {z_{i} } \right)} \right] = \left[ {\mu_{N}^{q} \left( {z_{i} } \right),1 - \nu_{N}^{q} \left( {z_{i} } \right)} \right]\,. $$

Thus, $OBPI_{M} \left( {z_{i} } \right)$ and $OBPI_{N} \left( {z_{i} } \right)$ are subintervals in $\left[ {0,\,1} \right]$. We then define the $OBPI_{M}$ of the q-ROFSs $M$ and $N$ as:

$$ OBPI_{M} = \left\{ {OBPI_{M} \left( {z_{i} } \right):z_{i} \in \tilde{Z}} \right\}\,\,{\text{and}}\,\,OBPI_{N} = \left\{ {OBPI_{N} \left( {z_{i} } \right):z_{i} \in \overset{\lower0.5em\hbox{$\smash{\scriptscriptstyle\frown}$}}{Z} } \right\}. $$

$$\overset{\lower0.5em\hbox{$\smash{\scriptscriptstyle\frown}$}}{H} \left( {OBPI_{M} \left( {z_{i} } \right),\,\,OBPI_{N} \left( {z_{i} } \right)} \right)$$ is said to be Hausdorff distance between $$OBPI_{M} \left( {z_{i} } \right)\,\,$$ and $$OBPI_{N} \left( {z_{i} } \right)$$ respectively. The Hausdorff distance between two orthoparian belief and plausible fuzzy intervals $$OBPI_{M} \,$$ and $$OBPI_{N} \,$$ will be,

8 $$ d_{BPH} \left( {OBPI_{M} ,\,OBPI_{N} } \right) = \frac{1}{n}\sum\limits_{i = 1}^{n} {\overset{\lower0.5em\hbox{$\smash{\scriptscriptstyle\frown}$}}{H} \left( {OBPI_{M} \left( {z_{i} } \right),\,\,OBPI_{N} \left( {z_{i} } \right)} \right)} .\, $$

Now, the distance $d$ on $OBPIs$ is axiomatically defined below.

### Definition 9

Consider $OBPI_{M}$,$OBPI_{N}$ and $OBPI_{O}$ be the three $OBPIs$ corresponding to three q-ROFSs $M$_,_ $N$ and ${\text{O}}$ on $\tilde{Z}$. Then the distance $d$ of Eq. (8) on $OBPIs$ is said to be distance if it fulfills the below axioms:

$$ \left( {d_{1} } \right)\,\,\,\,\,\,\,\,0 \le d\left( {OBPI_{M} ,OBPI_{N} } \right) \le 1; $$

$$ \left( {d_{2} } \right)\,\,\,\,\,\,\,\,\,\,d\left( {OBPI_{M} ,OBPI_{N} } \right) = 0\,\,iff\,\,OBPI_{M} = OBPI_{N} ; $$

$$ \left( {d_{3} } \right)\,\,\,\,\,\,\,\,\,\,\,d\left( {OBPI_{M} ,OBPI_{N} } \right) = d\left( {OBPI_{N} ,OBPI_{M} } \right); $$

$\left( {d_{4} } \right)\,$ $\,\,\,If\,\,OBPI_{M} \le OBPI_{N} \, \le \,OBPI_{O} \,$ be any three orthoparian belief-plausibility intervals, then, $d\left( {OBPI_{M} ,OBPI_{N} } \right) \le d\left( {OBPI_{N} ,OBPI_{O} } \right)$, $d\left( {OBPI_{M} ,OBPI_{O} } \right) \le d\left( {OBPI_{N} ,OBPI_{O} } \right);$

$\left( {d_{5} } \right)\,\,\,\,\,\,\,\,\,\,If\,\,OBPI_{M} ,OBPI_{N} \,{\text{and}}\,OBPI_{O} \,$ be any three orthoparian belief-plausibility intervals, then, $d\left( {OBPI_{M} ,OBPI_{O} } \right) \le d\left( {OBPI_{M} ,OBPI_{N} } \right) + d\left( {OBPI_{N} ,OBPI_{O} } \right)$.

To check the validity of proposed distance measure, we need to prove all the above axioms for orthoparian belief-plausibility intervals.

### Theorem 2

Let $\tilde{Z} = \left\{ {z_{1} ,z_{2} ,...,z_{n} } \right\}$ be universe of discourses, then the proposed distance measure $d_{BPH} \left( {OBPI_{M} ,OBPI_{N} } \right)$ for belief-plausibility intervals in q-ROFSs $M$ and $N\,$ satisfy all axioms of Definition 9.

### Axiom 1

$$0 \le d\left( {OBPI_{M} ,OBPI_{N} } \right) \le 1$$.

### Proof

Consider $$OBPI_{M} \,{\text{and}}\,OBPI_{N}$$ are two belief-plausibility intervals on q-ROFSs in the universe of discourse $$\tilde{Z} = \left\{ {z_{1} ,z_{2} ,...,z_{n} } \right\}.$$ According to the proposed distance measure $$d_{BPH} \left( {OBPI_{M} ,\,\,OBPI_{N} } \right) = \frac{1}{n}\sum\limits_{i = 1}^{n} {\overset{\lower0.5em\hbox{$\smash{\scriptscriptstyle\frown}$}}{H} \left( {OBPI_{M} \left( {z_{i} } \right),\,\,\,OBPI_{N} \left( {z_{i} } \right)} \right)} ,$$ where $$0 \le d_{BPH} \left( {OBPI_{M} ,OBPI_{N} } \right) \le 1\,$$. So, axiom 1 is proved because of absolute values.

### Axiom 2

$$d\left( {OBPI_{M} ,OBPI_{N} } \right) = 0\,\,\,\,iff\,\,OBPI_{M} = OBPI_{N}$$.

### Proof

If $$OBPI_{M} = OBPI_{N}$$ then $$Bl_{M} \left( {z_{i} } \right) = Bl_{N} \left( {z_{i} } \right)$$ and $$Pl_{M} \left( {z_{i} } \right) = Pl_{N} \left( {z_{i} } \right)$$ thus $$d_{BPH} \left( {OBPI_{M} ,OBPI_{N} } \right) = 0$$. Conversely, if $$d_{BPH} \left( {OBPI_{M} ,OBPI_{N} } \right) = 0,\,\,\forall \,z_{i} \in \overset{\lower0.5em\hbox{$\smash{\scriptscriptstyle\frown}$}}{G}$$. $$\left| {Bl_{M} \left( {z_{i} } \right) - Bl_{N} \left( {z_{i} } \right)} \right| = 0$$ and $$\,\left| {Pl_{M} \left( {z_{i} } \right) - Pl_{N} \left( {z_{i} } \right)} \right| = 0\,\,\,\,$$, since $$Bl_{M} \left( {z_{i} } \right) = Bl_{N} \left( {z_{i} } \right)\,\,$$ and $$Pl_{M} \left( {z_{i} } \right) = Pl_{N} \left( {z_{i} } \right)$$. Thus, axiom 2 is satisfied.

### Axiom 3

$$d\left( {OBPI_{M} ,OBPI_{N} } \right) = d\left( {OBPI_{N} ,OBPI_{M} } \right)$$.

### Proof

Since, $$\max \left\{ {\left| {Bl_{M} \left( {z_{i} } \right) - Bl_{N} \left( {z_{i} } \right)} \right|,\left| {Pl_{M} \left( {z_{i} } \right) - Pl_{N} \left( {z_{i} } \right)} \right|} \right\} = \max \left\{ {\left| {Bl_{N} \left( {z_{i} } \right) - Bl_{M} \left( {z_{i} } \right)} \right|,Pl\left| {_{N} \left( {z_{i} } \right) - Pl_{M} \left( {z_{i} } \right)} \right|} \right\}$$, so, $$\overset{\lower0.5em\hbox{$\smash{\scriptscriptstyle\frown}$}}{H} \left( {OBPI_{M} ,OBPI_{N} } \right) = \overset{\lower0.5em\hbox{$\smash{\scriptscriptstyle\frown}$}}{H} \left( {OBPI_{N} ,OBPI_{M} } \right),\,\,\sum\limits_{i = 1}^{n} {\overset{\lower0.5em\hbox{$\smash{\scriptscriptstyle\frown}$}}{H} \left( {OBPI_{M} ,OBPI_{N} } \right)} = \sum\limits_{i = 1}^{n} {\overset{\lower0.5em\hbox{$\smash{\scriptscriptstyle\frown}$}}{H} \left( {OBPI_{N} ,OBPI_{M} } \right)} ,$$ hence, $$d_{BPH} \left( {OBPI_{M} ,OBPI_{N} } \right) = d_{BPH} \left( {OBPI_{N} ,OBPI_{M} } \right)$$. Thus axiom 3 is satisfied.

### Axiom 4

If $OBPI_{M} \le OBPI_{N} \, \le \,OBPI_{O} \,$ be any three orthoparian belief-plausibility intervals, then $d\left( {OBPI_{M} ,OBPI_{N} } \right) \le d\left( {OBPI_{N} ,OBPI_{O} } \right)$, $d\left( {OBPI_{M} ,OBPI_{O} } \right) \le d\left( {OBPI_{N} ,OBPI_{O} } \right)$.

### Proof

Now, $$OBPI_{M} \le OBPI_{N} \, \le OBPI_{O}$$ then, $$Bl_{M} \left( {z_{i} } \right) \le Bl_{N} \left( {z_{i} } \right) \le Bl_{O} \left( {z_{i} } \right)$$ and $$Pl_{M} \left( {z_{i} } \right) \ge Pl_{N} \left( {z_{i} } \right) \ge Pl_{O} \left( {z_{i} } \right)$$ thus, we have, $$\overset{\lower0.5em\hbox{$\smash{\scriptscriptstyle\frown}$}}{H} \left( {OBPI_{M} ,OBPI_{N} } \right) = \max \left\{ {\left| {Bl_{M} \left( {z_{i} } \right) - Bl_{N} \left( {z_{i} } \right)} \right|,\left| {Pl_{M} \left( {z_{i} } \right) - Pl_{N} \left( {z_{i} } \right)} \right|} \right\}$$ and $$\overset{\lower0.5em\hbox{$\smash{\scriptscriptstyle\frown}$}}{H} \left( {OBPI_{N} ,OBPI_{O} } \right) = \max \left\{ {\left| {Bl_{N} \left( {z_{i} } \right) - Bl_{O} \left( {z_{i} } \right)} \right|,\left| {Pl_{N} \left( {z_{i} } \right) - Pl_{O} \left( {z_{i} } \right)} \right|} \right\}$$ hence $$\overset{\lower0.5em\hbox{$\smash{\scriptscriptstyle\frown}$}}{H} \left( {OBPI_{M} ,OBPI_{O} } \right) = \max \left\{ {\left| {Bl_{M} \left( {z_{i} } \right) - Bl_{O} \left( {z_{i} } \right)} \right|,\left| {Pl_{M} \left( {z_{i} } \right) - Pl_{O} \left( {z_{i} } \right)} \right|} \right\}$$ which implies that, if $$\left| {Bl_{M} \left( {z_{i} } \right) - Bl_{O} \left( {z_{i} } \right)} \right| \ge \left| {Pl_{M} \left( {z_{i} } \right) - Pl_{O} \left( {z_{i} } \right)} \right|$$ then $$\overset{\lower0.5em\hbox{$\smash{\scriptscriptstyle\frown}$}}{H} \left( {OBPI_{M} ,OBPI_{O} } \right) = \left| {Bl_{M} \left( {z_{i} } \right) - Bl_{O} \left( {z_{i} } \right)} \right|$$ but we have $$\left| {Pl_{M} \left( {z_{i} } \right) - Pl_{N} \left( {z_{i} } \right)} \right| \le \left| {Pl_{M} \left( {z_{i} } \right) - Pl_{O} \left( {z_{i} } \right)} \right| \le \left| {Bl_{M} \left( {z_{i} } \right) - Bl_{O} \left( {z_{i} } \right)} \right|$$ and $$\left| {Pl_{N} \left( {z_{i} } \right) - Pl_{O} \left( {z_{i} } \right)} \right| \le \left| {Pl_{M} \left( {z_{i} } \right) - Pl_{O} \left( {z_{i} } \right)} \right| \le \left| {Bl_{M} \left( {z_{i} } \right) - Bl_{O} \left( {z_{i} } \right)} \right|\,.$$

On the contrary we have,$\left| {Bl_{M} \left( {z_{i} } \right) - Bl_{N} \left( {z_{i} } \right)} \right| \le \left| {Bl_{N} \left( {z_{i} } \right) - Bl_{O} \left( {z_{i} } \right)} \right|\,$ and $\left| {Bl_{N} \left( {z_{i} } \right) - Bl_{O} \left( {z_{i} } \right)} \right| \le \left| {Bl_{M} \left( {z_{i} } \right) - Bl_{O} \left( {z_{i} } \right)} \right|$.

By combining we get, $$\overset{\lower0.5em\hbox{$\smash{\scriptscriptstyle\frown}$}}{H} \left( {OBPI_{M} ,OBPI_{N} } \right) \le \overset{\lower0.5em\hbox{$\smash{\scriptscriptstyle\frown}$}}{H} \left( {OBPI_{M} ,OBPI_{O} } \right)$$ and $$\overset{\lower0.5em\hbox{$\smash{\scriptscriptstyle\frown}$}}{H} \left( {OBPI_{N} ,OBPI_{O} } \right) \le \overset{\lower0.5em\hbox{$\smash{\scriptscriptstyle\frown}$}}{H} \left( {OBPI_{M} ,OBPI_{O} } \right)$$ which implies that, $$d_{BPH} \left( {OBPI_{M} ,OBPI_{N} } \right) \le d_{BPH} \left( {OBPI_{M} ,OBPI_{O} } \right)$$ and $$d_{BPH} \left( {OBPI_{N} ,OBPI_{O} } \right) \le d_{BPH} \left( {OBPI_{M} ,OBPI_{O} } \right)$$.

$$\overset{\lower0.5em\hbox{$\smash{\scriptscriptstyle\frown}$}}{H} \left( {OBPI_{M} ,OBPI_{N} } \right) \le \overset{\lower0.5em\hbox{$\smash{\scriptscriptstyle\frown}$}}{H} \left( {OBPI_{M} ,OBPI_{O} } \right)\,{\text{and}}\,\,\overset{\lower0.5em\hbox{$\smash{\scriptscriptstyle\frown}$}}{H} \left( {OBPI_{N} ,OBPI_{O} } \right) \le \overset{\lower0.5em\hbox{$\smash{\scriptscriptstyle\frown}$}}{H} \left( {OBPI_{M} ,OBPI_{O} } \right),$$ implies that $$d_{BPH} \left( {OBPI_{M} ,OBPI_{N} } \right) \le d_{BPH} \left( {OBPI_{M} ,OBPI_{O} } \right)$$. and $$d_{BPH} \left( {OBPI_{N} ,OBPI_{O} } \right) \le d_{BPH} \left( {OBPI_{M} ,OBPI_{O} } \right)$$ implies that, if $$\left| {Bl_{M} \left( {z_{i} } \right) - Bl_{O} \left( {z_{i} } \right)} \right| \le \left| {Pl_{M} \left( {z_{i} } \right) - Pl_{O} \left( {z_{i} } \right)} \right|\,$$ then $$\,\overset{\lower0.5em\hbox{$\smash{\scriptscriptstyle\frown}$}}{H} \left( {OBPI_{M} ,OBPI_{O} } \right) = \left| {Pl_{M} \left( {z_{i} } \right) - Pl_{O} \left( {z_{i} } \right)} \right|\,$$, but we have, $$\,\left| {Bl_{M} \left( {z_{i} } \right) - Bl_{N} \left( {z_{i} } \right)} \right| \le \left| {Bl_{M} \left( {z_{i} } \right) - Bl_{O} \left( {z_{i} } \right)} \right| \le \left| {Pl_{M} \left( {z_{i} } \right) - Pl_{O} \left( {z_{i} } \right)} \right|\,\,$$ and $$\left| {Bl_{N} \left( {z_{i} } \right) - Bl_{O} \left( {z_{i} } \right)} \right| \le \left| {Bl_{M} \left( {z_{i} } \right) - Bl_{O} \left( {z_{i} } \right)} \right| \le \left| {Pl_{M} \left( {z_{i} } \right) - Pl_{O} \left( {z_{i} } \right)} \right|$$. On the contrary we have, $$\left| {Pl_{M} \left( {z_{i} } \right) - Pl_{N} \left( {z_{i} } \right)} \right| \le \left| {Pl_{M} \left( {z_{i} } \right) - Pl_{O} \left( {z_{i} } \right)} \right|\,\,$$ and $$\left| {Pl_{N} \left( {z_{i} } \right) - Pl_{O} \left( {z_{i} } \right)} \right| \le \left| {Pl_{M} \left( {z_{i} } \right) - Pl_{O} \left( {z_{i} } \right)} \right|$$.

By combining we get $$\overset{\lower0.5em\hbox{$\smash{\scriptscriptstyle\frown}$}}{H} \left( {OBPI_{M} ,OBPI_{N} } \right) \le \overset{\lower0.5em\hbox{$\smash{\scriptscriptstyle\frown}$}}{H} \left( {OBPI_{M} ,OBPI_{O} } \right),$$ and $$\,\overset{\lower0.5em\hbox{$\smash{\scriptscriptstyle\frown}$}}{H} \left( {OBPI_{M} ,OBPI_{O} } \right) \le \overset{\lower0.5em\hbox{$\smash{\scriptscriptstyle\frown}$}}{H} \left( {OBPI_{M} ,OBPI_{O} } \right)$$ implies that, $$d_{BPH} \left( {OBPI_{M} ,OBPI_{N} } \right) \le d_{BPH} \left( {OBPI_{M} ,OBPI_{O} } \right)$$ and $$d_{BPH} \left( {OBPI_{N} ,OBPI_{O} } \right) \le d_{BPH} \left( {OBPI_{M} ,OBPI_{O} } \right)$$. So, this proves the axiom 4.

### Axiom 5

$If\,\,OBPI_{M} ,OBPI_{N} \,{\text{and}}\,OBPI_{O} \,$ be any three orthoparian belief-plausibility intervals, then $d\left( {OBPI_{M} ,OBPI_{O} } \right) \le d\left( {OBPI_{M} ,OBPI_{N} } \right) + d\left( {OBPI_{N} ,OBPI_{O} } \right)$.

### Proof

To prove the triangular inequality of $$\left( {d_{5} } \right)$$, let $$OBPI_{M} ,OBPI_{N} \,$$ and $$OBPI_{O} \,$$ be the three belief and plausibility intervals. If $$\left| {Bl_{M} \left( {z_{i} } \right) - Bl_{O} \left( {z_{i} } \right)} \right| \ge \left| {Pl_{M} \left( {z_{i} } \right) - Pl_{O} \left( {z_{i} } \right)} \right|\,$$ then, $$\,\overset{\lower0.5em\hbox{$\smash{\scriptscriptstyle\frown}$}}{H} \left( {OBPI_{M} ,OBPI_{O} } \right) = \left| {Bl_{M} \left( {z_{i} } \right) - Bl_{O} \left( {z_{i} } \right)} \right|$$. So, $$\overset{\lower0.5em\hbox{$\smash{\scriptscriptstyle\frown}$}}{H} \left( {OBPI_{M} ,OBPI_{O} } \right) = \left| {Bl_{M} \left( {z_{i} } \right) - Bl_{N} \left( {z_{i} } \right) + Bl_{N} \left( {z_{i} } \right) - Bl_{O} \left( {z_{i} } \right)} \right|.$$

Thus, $$\overset{\lower0.5em\hbox{$\smash{\scriptscriptstyle\frown}$}}{H} \left( {OBPI_{M} ,OBPI_{O} } \right) \le \left| {Bl_{M} \left( {z_{i} } \right) - Bl_{N} \left( {z_{i} } \right)} \right| + \left| {Bl_{N} \left( {z_{i} } \right) - Bl_{O} \left( {z_{i} } \right)} \right|$$ and $$\overset{\lower0.5em\hbox{$\smash{\scriptscriptstyle\frown}$}}{H} \left( {OBPI_{M} ,OBPI_{O} } \right) \le d\left( {OBPI_{M} ,OBPI_{N} } \right) + d\left( {OBPI_{N} ,OBPI_{O} } \right)$$ where, $$d_{BPH} \left( {OBPI_{M} ,OBPI_{N} } \right) = max\left\{ {\left| {Bl_{M} \left( {z_{i} } \right) - Bl_{N} \left( {z_{i} } \right)} \right|,\left| {Pl_{M} \left( {z_{i} } \right) - Pl_{N} \left( {z_{i} } \right)} \right|} \right\}$$ and $$d_{BPH} \left( {OBPI_{N} ,OBPI_{O} } \right) = max\left\{ {\left| {Bl_{N} \left( {z_{i} } \right) - Bl_{O} \left( {z_{i} } \right)} \right|,\left| {Pl_{N} \left( {z_{i} } \right) - Pl_{O} \left( {z_{i} } \right)} \right|} \right\}$$. Thus, $$\overset{\lower0.5em\hbox{$\smash{\scriptscriptstyle\frown}$}}{H} \left( {OBPI_{M} ,OBPI_{O} } \right) \le \overset{\lower0.5em\hbox{$\smash{\scriptscriptstyle\frown}$}}{H} \left( {OBPI_{M} ,OBPI_{N} } \right) + \overset{\lower0.5em\hbox{$\smash{\scriptscriptstyle\frown}$}}{H} \left( {OBPI_{N} ,OBPI_{O} } \right)$$ implies that $$d_{BPH} \left( {OBPI_{M} ,OBPI_{O} } \right) \le d_{BPH} \left( {OBPI_{M} ,OBPI_{N} } \right) + d_{BPH} \left( {OBPI_{N} ,OBPI_{O} } \right).$$

If $$\left| {Bl_{M} \left( {z_{i} } \right) - Bl_{O} \left( {z_{i} } \right)} \right| \le \left| {Pl_{M} \left( {z_{i} } \right) - Pl_{O} \left( {z_{i} } \right)} \right|$$, then $$\overset{\lower0.5em\hbox{$\smash{\scriptscriptstyle\frown}$}}{H} \left( {OBPI_{M} ,OBPI_{O} } \right) = \left| {Pl_{M} \left( {z_{i} } \right) - Pl_{O} \left( {z_{i} } \right)} \right|$$.

So, $$\overset{\lower0.5em\hbox{$\smash{\scriptscriptstyle\frown}$}}{H} \left( {OBPI_{M} ,OBPI_{O} } \right) = \left| {Pl_{M} \left( {z_{i} } \right) - Pl_{N} \left( {z_{i} } \right) + Pl_{N} \left( {z_{i} } \right) - Pl_{O} \left( {z_{i} } \right)} \right|$$

$$ \overset{\lower0.5em\hbox{$\smash{\scriptscriptstyle\frown}$}}{H} \left( {OBPI_{M} ,OBPI_{O} } \right) \le \left| {Pl_{M} \left( {z_{i} } \right) - Pl_{N} \left( {z_{i} } \right)} \right| + \left| {Pl_{N} \left( {z_{i} } \right) - Pl_{O} \left( {z_{i} } \right)} \right| \le d\left( {OBPI_{M} ,OBPI_{N} } \right) + d\left( {OBPI_{N} ,OBPI_{O} } \right). $$

where $$d\left( {OBPI_{M} ,OBPI_{N} } \right) = max\left\{ {\left| {Bl_{M} \left( {z_{i} } \right) - Bl_{N} \left( {z_{i} } \right)} \right|,\left| {Pl_{M} \left( {z_{i} } \right) - Pl_{N} \left( {z_{i} } \right)} \right|} \right\}$$ and $$d\left( {OBPI_{N} ,OBPI_{O} } \right) = max\left\{ {\left| {Bl_{N} \left( {z_{i} } \right) - Bl_{O} \left( {z_{i} } \right)} \right|,\left| {Pl_{N} \left( {z_{i} } \right) - Pl_{O} \left( {z_{i} } \right)} \right|} \right\}$$. Hence, $$\overset{\lower0.5em\hbox{$\smash{\scriptscriptstyle\frown}$}}{H} \left( {OBPI_{M} ,OBPI_{O} } \right) \le \overset{\lower0.5em\hbox{$\smash{\scriptscriptstyle\frown}$}}{H} \left( {OBPI_{M} ,OBPI_{N} } \right) + \overset{\lower0.5em\hbox{$\smash{\scriptscriptstyle\frown}$}}{H} \left( {OBPI_{N} ,OBPI_{O} } \right)$$ implies that, $$d_{BPH} \left( {OBPI_{M} ,OBPI_{O} } \right) \le d_{BPH} \left( {OBPI_{M} ,OBPI_{N} } \right) + d_{BPH} \left( {OBPI_{N} ,OBPI_{O} } \right)$$. Thus, all axioms are proved. □

### Theorem 3

Consider $OBPI_{M} \,$ and $OBPI_{N}$ are two belief-plausibility intervals on q-ROFSs in the universe of discourse $\tilde{Z} = \left\{ {z_{1} ,z_{2} ,...,z_{n} } \right\}$ then the proposed distance fulfills the following properties:

1. $$d_{BPH} \left( {OBPI_{M} ,OBPI_{N} } \right) = d_{BPH} \left( {OBPI_{M} \cap OBPI_{N} ,OBPI_{M} \cup OBPI_{N} } \right)$$.

2. $$d_{BPH} \left( {OBPI_{M} ,OBPI_{M} \cap OBPI_{N} } \right) = d_{BPH} \left( {OBPI_{N} ,OBPI_{M} \cup OBPI_{N} } \right)$$.

3. $$d_{BPH} \left( {OBPI_{M} ,OBPI_{M} \cup OBPI_{N} } \right) = d_{BPH} \left( {OBPI_{N} ,OBPI_{M} \cap OBPI_{N} } \right)$$.

## Similarity measures of q-ROFSs for belief and plausibility intervals

Similarity measures are very important tools to distinguish two different sets or objects. Similarity measure has numerous applications in many fields including pattern recognition, clustering and MCCDM^22,53,54^. Distance and similarity measures are commonly considered as dual concepts. So we can also find the similarity directly if the distance is given from the relation $${\text{similarity}}\,{ = 1 - }\,{\text{distance}}$$. Hence, the Hausdorff distance between two $$OBPIs,$$$$OBPI_{M}$$ and $$OBPI_{N}$$ of q-ROFSs is used to define similarities between them. Let $$f$$ be a monotone decreasing function and $$OBPI_{M}$$ and $$OBPI_{N}$$ be two $$OBPIs$$. Since $$0 \le d_{BPH} \left( {OBPI_{M} ,OBPI_{N} } \right) \le 1,$$$$f\left( 1 \right) \le f\left( {d_{BPH} \left( {OBPI_{M} ,OBPI_{N} } \right)} \right) \le f\left( 0 \right),$$ $$f\left( 1 \right) \le f\left( {d_{BPH} \left( {OBPI_{M} ,OBPI_{N} } \right)} \right) \le f\left( 0 \right)$$ which implies that $$f\left( 1 \right) \le f\left( {d_{BPH} \left( {OBPI_{M} ,OBPI_{N} } \right)} \right) \le f\left( 0 \right)$$. So, the similarity measure among $$OBPI_{M}$$ and $$OBPI_{N}$$ will be,

9 $$ S\left( {OBPI_{M} ,OBPI_{N} } \right) = \frac{{f\left( {d_{BPH} \left( {OBPI_{M} ,OBPI_{N} } \right)} \right) - f\left( 1 \right)}}{f\left( 0 \right) - f\left( 1 \right)}\, $$

After selecting suitable functions, we get various similarity measures with the help of above Eq. (9). First we select the simple linear function $f\left( x \right) = 1 - x$. From Eq. (9), the similarity measure among $OBPI_{M}$ and $OBPI_{N}$ of q-ROFSs will be.

10 $$ S_{L} \left( {OBPI_{M} ,OBPI_{N} } \right) = 1 - d_{BPH} \left( {OBPI_{M} ,OBPI_{N} } \right). $$

Again, we select the simple function $f\left( x \right) = \frac{1}{1 + x}$ so, the similarity measure among $OBPI_{M}$ and $OBPI_{N}$ of q-ROFS can be defined as follows:

11 $$ S_{Q} \left( {OBPI_{M} ,OBPI_{N} } \right) = \frac{{1 - d_{BPH} \left( {OBPI_{M} ,OBPI_{N} } \right)}}{{1 + d_{BPH} \left( {OBPI_{M} ,OBPI_{N} } \right)}}\, $$

Also, we select the well-known function $f\left( x \right) = e^{ - x}$. So, the similarity measure between $OBPI_{M}$ and $OBPI_{N}$ of q-ROFS is constructed as:

12 $$ S_{E} \left( {OBPI_{M} ,OBPI_{N} } \right) = \frac{{e^{{ - d_{BPH} \left( {OBPI_{M} ,OBPI_{N} } \right)}} - e^{ - 1} }}{{1 - e^{ - 1} }} $$

Now, some numerical examples are presented to test the validity of suggested similarities of Eqs. (10)–(12).

### Example 2

Consider $\tilde{Z} = \left\{ {z_{1} ,z_{2} ,z_{3} } \right\}\,$ be a universal set and let we have any two q-ROFSs as: $M = \left\{ {\left\langle {z_{1} ,0.87,0.65} \right\rangle \left\langle {z_{2} ,0.9,0.5} \right\rangle } \right\}\,\,{\text{and}}\,N = \left\{ {\left\langle {z_{1} ,0.6,0.9} \right\rangle \left\langle {z_{2} ,0.81,0.7} \right\rangle } \right\}$. We have to solve proposed similarity measure between belief-plausibility of q-ROFSs.

$$ OBPI_{M} = \left\{ {\left\langle {z_{1} ,\left[ {0.658,0.725} \right]} \right\rangle ,\left\langle {z_{2} ,\left[ {0.729,0.125} \right]} \right\rangle } \right\} $$

$$OBPI_{N} = \left\{ {\left\langle {z_{1} ,\left[ {0.216,0.729} \right]} \right\rangle ,\left\langle {z_{2} ,\left[ {0.531,0.343} \right]} \right\rangle } \right\}$$,

$$ \overset{\lower0.5em\hbox{$\smash{\scriptscriptstyle\frown}$}}{H} \left( {OBPI_{M} ,OBPI_{N} } \right) = \left\{ {\left\langle {z_{1} ,0.442} \right\rangle ,\left\langle {z_{2} ,0.218} \right\rangle } \right\} $$

$$d\left( {OBPI_{M} ,OBPI_{N} } \right) = \frac{1}{n}\sum\limits_{i = 1}^{n} {\overset{\lower0.5em\hbox{$\smash{\scriptscriptstyle\frown}$}}{H} \left( {OBPI_{M} ,OBPI_{N} } \right)} = 0.333$$.

By using Eq. (10)–(12), we have,

$$S_{L} \left( {OBPI_{M} ,OBPI_{N} } \right) = 0.667$$; $$S_{Q} \left( {OBPI_{M} ,OBPI_{N} } \right) = 0.5003$$; $$S_{E} \left( {OBPI_{M} ,OBPI_{N} } \right) = 0.5518$$.

### Application in pattern recognition

Pattern recognition focuses on how computer programs can recognize various patterns. It is used to classify or categorize these patterns and make judgments regarding their classes. Due to its expanding relevance in several domains, this has been a major and compelling study topic for almost five decades. We provide an example of pattern recognition by employing the proposed similarities of Eqs. (10)–(12) below.

#### Example 3

Assume that we have three different classes of viruses with specific characteristics. These classes are expressed by three q-ROFSs $M_{1}$, $M_{2}$ and $M_{3}$ respectively, which can be represented as follows:

$M_{1} = \left\{ {\left\langle {z_{1} ,0.4,0.8} \right\rangle ,\left\langle {z_{2} ,0.5,0.7} \right\rangle ,\left\langle {z_{3} ,0.6,0.5} \right\rangle } \right\}$, $M_{2} = \left\{ {\left\langle {z_{1} ,0.2,0.9} \right\rangle ,\left\langle {z_{2} ,0.4,0.7} \right\rangle ,\left\langle {z_{3} ,0.6,0.7} \right\rangle } \right\}$ and $M_{3} = \left\{ {\left\langle {z_{1} ,0.7,0.6} \right\rangle ,\left\langle {z_{2} ,0.9,0.8} \right\rangle ,\left\langle {z_{3} ,0.8,0.6} \right\rangle } \right\}$ on universe of discourses $Z = \left\{ {z_{1} ,z_{2} ,z_{3} } \right\}$**.**

Assume that another class of virus $N = \left\{ {\left\langle {z_{1} ,0.5,0.7} \right\rangle ,\left\langle {z_{2} ,0.5,0.6} \right\rangle ,\left\langle {z_{3} ,0.7,0.5} \right\rangle } \right\}$ is given as a sample. The basic goal is to interpret the idea of the given patterns and demonstrate which class of virus the sample $N$ belongs to. The corresponding OBPIs $OBPI_{{M_{1} }}$, $OBPI_{{M_{2} }}$, $OBPI_{{M_{3} }}$ and $OBPI_{N}$ of q-ROFSs $M_{1}$, $M_{2}$, $M_{3}$ and $N$ respectively are:

$$ OBPI_{{M_{1} }} = \left\{ {\left\langle {z_{1} ,0.4,0.2} \right\rangle ,\left\langle {z_{2} ,0.5,0.3} \right\rangle ,\left\langle {z_{3} ,0.6,0.5} \right\rangle } \right\}; $$

$$ OBPI_{{M_{2} }} = \left\{ {\left\langle {z_{1} ,0.2,0.1} \right\rangle ,\left\langle {z_{2} ,0.6,0.3} \right\rangle ,\left\langle {z_{3} ,0.6,0.3} \right\rangle } \right\}; $$

$$ OBPI_{{M_{3} }} = \left\{ {\left\langle {z_{1} ,0.7,0.4} \right\rangle ,\left\langle {z_{2} ,0.9,0.8} \right\rangle ,\left\langle {z_{3} ,0.8,0.6} \right\rangle } \right\}; $$

$$ OBPI_{N} = \left\{ {\left\langle {z_{1} ,0.5,0.3} \right\rangle ,\left\langle {z_{2} ,0.5,0.4} \right\rangle ,\left\langle {z_{3} ,0.7,0.3} \right\rangle } \right\}. $$

By utilizing the proposed similarity measures of Eqs. (8)–(10), we have,

$$S_{L} \left( {OBPI_{{M_{1} }} ,OBPI_{N} } \right) = 0.859,$$$$S_{L} \left( {OBPI_{{M_{2} }} ,OBPI_{N} } \right) = 0.756,$$ $$S_{L} \left( {OBPI_{{M_{3} }} ,OBPI_{N} } \right) = 0.670,$$

$$S_{Q} \left( {OBPI_{{M_{1} }} ,OBPI_{N} } \right) = 0.753,$$ $$S_{Q} \left( {OBPI_{{M_{2} }} ,OBPI_{N} } \right) = 0.608,$$ $$S_{Q} \left( {OBPI_{{M_{3} }} ,OBPI_{N} } \right) = 0.401,$$

$$S_{E} \left( {OBPI_{{M_{1} }} ,OBPI_{N} } \right) = 0.792,$$ $$S_{E} \left( {OBPI_{{M_{2} }} ,OBPI_{N} } \right) = 0.657,$$ $$S_{E} \left( {OBPI_{{M_{3} }} ,OBPI_{N} } \right) = 0.555.$$

**Figure 2:**
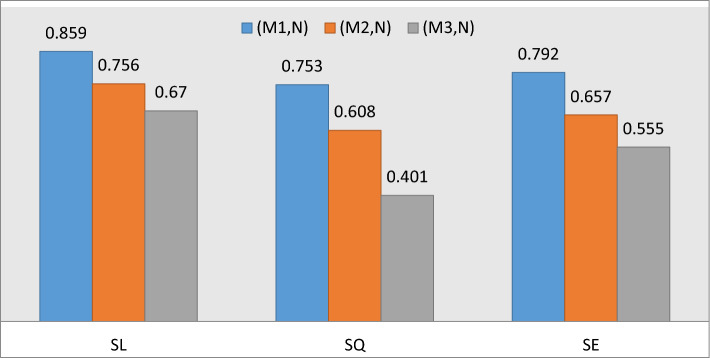
Graph of similarity between the patterns $M_{1}$, $M_{2}$, $M_{3}$ and sample $N$.

These numerical computations depicts that the similarity among the OBPI pattern $OBPI_{{M_{1} }}$ and the OBPI sample $OBPI_{N}$ is larger as compare to similarity between OBPI patterns $OBPI_{{M_{2} }}$ and $OBPI_{{M_{3} }}$ respectively to the sample $OBPI_{N}$. Thus, the given sample $OBPI_{N}$ resembles with OBPI pattern $OBPI_{{M_{1} }}$ based on the highest degree of similarity among two OBPI patterns. In other words, the sample $N$ resembles with pattern $M_{1}$ as compare to patterns $M_{2}$ and $M_{3}$ respectively which are represented in Fig. 2.

From the above figure, it is clear that the sample $N$ resembles with pattern $M_{1}$.

### Application in clustering

Clustering is a type of unsupervised machine learning technique that involves grouping together data points based on their similarity. Clustering algorithms are used to partition a set of data points into clusters, where the points within each cluster are more similar to each other than they are to points in other clusters. Now, we present the application of proposed similarity measure Eq. (10) in clustering. The steps towards clustering are given as follows:

Choose the function *f* using Eq. (10) and analyze $S\left( {OBPI_{{M_{i} }} ,OBPI_{{M_{j} }} } \right)$ and develop the Pythagorean similarity matrix.

(i) The satisfaction level is denoted by $\,\tau \in \left[ {0,1} \right]$.

Let $$\zeta_{ij}^{\tau } = \,\left\{ \begin{gathered} 1,\,\,S\left( {OBPI_{{M_{i} }} ,OBPI_{{M_{j} }} } \right)\,\, \ge \,\,\,\tau \hfill \\ 0,\,\,S\left( {OBPI_{{M_{i} }} ,OBPI_{{M_{j} }} } \right)\, < \,\,\tau \hfill \\ \end{gathered} \right.,$$ and then develop the $\tau$-cutting matrix $D_{\tau } = \left( {\zeta_{ij}^{\tau } } \right)_{n \times n}$.

(ii) Obtain the corresponding clustering result based on $D_{\tau } = \left( {\zeta_{ij}^{\tau } } \right)_{n \times n}$.

#### Example 4

For better evaluation of different medicines $M = \left\{ {M_{1} ,M_{2} ,...,M_{8} } \right\}$ available in drug stores, we set four different criterias which are: Efficacy (C_1_), Side effects (C_2_), Availability (C_3_), Cost and condition (C_4_). The evaluation information is expressed by orthoparian fuzzy sets on a universe of discourse $\tilde{Z}$ as:

$$ M_{1} = \left\{ {\left( {z_{1} ,\,0.8,\,\,0.7} \right),\left( {z_{2} ,0.3,\,\,0.7} \right),\left( {z_{3} ,0.6,\,\,0.5} \right),\left( {z_{4} ,0.7,\,\,0.5} \right)} \right\}; $$

$$ M_{2} = \left\{ {\left( {z_{1} ,\,0.9,\,\,0.5} \right),\left( {z_{2} ,0.7,\,\,0.3} \right),\left( {z_{3} ,0.8,\,\,0.4} \right),\left( {z_{4} ,0.6,\,\,0.7} \right)} \right\}; $$

$$ M_{3} = \left\{ {\left( {z_{1} ,\,0.9,\,\,0.4} \right),\left( {z_{2} ,0.8,\,\,0.3} \right),\left( {z_{3} ,0.6,\,\,0.7} \right),\left( {z_{4} ,0.8,\,\,0.6} \right)} \right\}; $$

$$ M_{4} = \left\{ {\left( {z_{1} ,\,0.7,\,\,0.6} \right),\left( {z_{2} ,0.9,\,\,0.5} \right),\left( {z_{3} ,0.2,\,\,0.9} \right),\left( {z_{4} ,0.8,\,\,0.7} \right)} \right\}; $$

$$ M_{5} = \left\{ {\left( {z_{1} ,\,0.7,\,\,0.8} \right),\left( {z_{2} ,0.4,\,\,0.8} \right),\left( {z_{3} ,0.6,\,\,0.6} \right),\left( {z_{4} ,0.7,\,\,0.6} \right)} \right\}; $$

$$ M_{6} = \left\{ {\left( {z_{1} ,\,0.8,\,\,0.5} \right),\left( {z_{2} ,0.7,\,\,0.4} \right),\left( {z_{3} ,0.8,\,\,0.5} \right),\left( {z_{4} ,0.7,\,\,0.8} \right)} \right\}; $$

$$ M_{7} = \left\{ {\left( {z_{1} ,\,0.9,\,\,0.6} \right),\left( {z_{2} ,0.8,\,\,0.5} \right),\left( {z_{3} ,0.7,\,\,0.7} \right),\left( {z_{4} ,0.8,\,\,0.6} \right)} \right\}; $$

$$ M_{8} = \left\{ {\left( {z_{1} ,\,0.7,\,\,0.7} \right),\left( {z_{2} ,0.8,\,\,0.6} \right),\left( {z_{3} ,0.3,\,\,0.9} \right),\left( {z_{4} ,0.7,\,\,0.9} \right)} \right\}. $$

The corresponding OBPIs are constructed as:

$$ OBPI_{{M_{1} }} = \left\{ {\left( {z_{1} ,\,0.512,\,\,0.657} \right),\left( {z_{2} ,0.027,\,\,0.657} \right),\left( {z_{3} ,0.216,\,\,0.875} \right),\left( {z_{4} ,0.343,\,\,0.875} \right)} \right\}; $$

$$ OBPI_{{M_{2} }} = \left\{ {\left( {z_{1} ,\,0.729,\,\,0.875} \right),\left( {z_{2} ,0.343,\,\,0.973} \right),\left( {z_{3} ,0.512,\,\,0.936} \right),\left( {z_{4} ,0.216,\,\,0.657} \right)} \right\}; $$

$$ OBPI_{{M_{3} }} = \left\{ {\left( {z_{1} ,\,0.729,\,\,0.936} \right),\left( {z_{2} ,0.512,\,\,0.973} \right),\left( {z_{3} ,0.216,\,\,0.657} \right),\left( {z_{4} ,0.512,\,\,0.784} \right)} \right\}; $$

$$ OBPI_{{M_{4} }} = \left\{ {\left( {z_{1} ,\,0.343,\,\,0.784} \right),\left( {z_{2} ,0.729,\,\,0.875} \right),\left( {z_{3} ,0.008,\,\,0.271} \right),\left( {z_{4} ,0.512,\,\,0.657} \right)} \right\}; $$

$$ OBPI_{{M_{5} }} = \left\{ {\left( {z_{1} ,\,0.343,\,\,0.488} \right),\left( {z_{2} ,0.064,\,\,0.488} \right),\left( {z_{3} ,0.216,\,\,0.784} \right),\left( {z_{4} ,0.343,\,\,0.784} \right)} \right\}; $$

$$ OBPI_{{M_{6} }} = \left\{ {\left( {z_{1} ,\,0.512,\,\,0.875} \right),\left( {z_{2} ,0.343,\,\,0.936} \right),\left( {z_{3} ,0.512,\,\,0.875} \right),\left( {z_{4} ,0.343,\,\,0.488} \right)} \right\}; $$

$$ OBPI_{{M_{7} }} = \left\{ {\left( {z_{1} ,\,0.729,\,\,0.784} \right),\left( {z_{2} ,0.512,\,\,0.875} \right),\left( {z_{3} ,0.343,\,\,0.657} \right),\left( {z_{4} ,0.512,\,\,0.784} \right)} \right\}; $$

$$ OBPI_{{M_{8} }} = \left\{ {\left( {z_{1} ,\,0.343,\,\,0.657} \right),\left( {z_{2} ,0.512,\,\,0.784} \right),\left( {z_{3} ,0.027,\,\,0.271} \right),\left( {z_{4} ,0.343,\,\,0.271} \right)} \right\}. $$

Step 1: Calculate $S\left( {OBPI_{{M_{i} }} ,OBPI_{{M_{j} }} } \right)$ by Eq. (10) as:

$$ S\left( {OBPI_{{M_{j} }} ,OBPI_{{M_{j} }} } \right) = 1\,\,\,{\text{when}}\,\,j = 1,2,...,8 $$

$$S\left( {OBPI_{{M_{1} }} ,OBPI_{{M_{2} }} } \right) = S\left( {OBPI_{{M_{2} }} ,OBPI_{{M_{1} }} } \right) = 0.738$$, $$S\left( {OBPI_{{M_{1} }} ,OBPI_{{M_{3} }} } \right) = S\left( {OBPI_{{M_{3} }} ,OBPI_{{M_{1} }} } \right) = 0.712$$,

$$S\left( {OBPI_{{M_{1} }} ,OBPI_{{M_{4} }} } \right) = S\left( {OBPI_{{M_{4} }} ,OBPI_{{M_{1} }} } \right) = 0.577$$, $$S\left( {OBPI_{{M_{1} }} ,OBPI_{{M_{5} }} } \right) = S\left( {OBPI_{{M_{5} }} ,OBPI_{{M_{1} }} } \right) = 0.870$$,

$$S\left( {OBPI_{{M_{1} }} ,OBPI_{{M_{6} }} } \right) = S\left( {OBPI_{{M_{6} }} ,OBPI_{{M_{1} }} } \right) = 0.696$$, $$S\left( {OBPI_{{M_{1} }} ,OBPI_{{M_{7} }} } \right) = S\left( {OBPI_{{M_{7} }} ,OBPI_{{M_{1} }} } \right) = 0.728$$,

$$S\left( {OBPI_{{M_{1} }} ,OBPI_{{M_{8} }} } \right) = S\left( {OBPI_{{M_{8} }} ,OBPI_{{M_{1} }} } \right) = 0.534$$, $$S\left( {OBPI_{{M_{2} }} ,OBPI_{{M_{3} }} } \right) = S\left( {OBPI_{{M_{3} }} ,OBPI_{{M_{2} }} } \right) = 0.794$$,

$$S\left( {OBPI_{{M_{2} }} ,OBPI_{{M_{4} }} } \right) = S\left( {OBPI_{{M_{4} }} ,OBPI_{{M_{2} }} } \right) = 0.567$$, $$S\left( {OBPI_{{M_{2} }} ,OBPI_{{M_{5} }} } \right) = S\left( {OBPI_{{M_{5} }} ,OBPI_{{M_{2} }} } \right) = 0.676$$,

$$S\left( {OBPI_{{M_{2} }} ,OBPI_{{M_{6} }} } \right) = S\left( {OBPI_{{M_{6} }} ,OBPI_{{M_{2} }} } \right) = 0.879$$, $$S\left( {OBPI_{{M_{2} }} ,OBPI_{{M_{7} }} } \right) = S\left( {OBPI_{{M_{7} }} ,OBPI_{{M_{2} }} } \right) = 0.791$$,

$$S\left( {OBPI_{{M_{2} }} ,OBPI_{{M_{8} }} } \right) = S\left( {OBPI_{{M_{8} }} ,OBPI_{{M_{2} }} } \right) = 0.593$$, $$S\left( {OBPI_{{M_{3} }} ,OBPI_{{M_{4} }} } \right) = S\left( {OBPI_{{M_{4} }} ,OBPI_{{M_{3} }} } \right) = 0.721$$,

$$S\left( {OBPI_{{M_{3} }} ,OBPI_{{M_{5} }} } \right) = S\left( {OBPI_{{M_{5} }} ,OBPI_{{M_{3} }} } \right) = 0.693$$, $$S\left( {OBPI_{{M_{3} }} ,OBPI_{{M_{6} }} } \right) = S\left( {OBPI_{{M_{6} }} ,OBPI_{{M_{3} }} } \right) = 0.755$$,

$$S\left( {OBPI_{{M_{3} }} ,OBPI_{{M_{7} }} } \right) = S\left( {OBPI_{{M_{7} }} ,OBPI_{{M_{3} }} } \right) = 0.906$$, $$S\left( {OBPI_{{M_{3} }} ,OBPI_{{M_{8} }} } \right) = S\left( {OBPI_{{M_{8} }} ,OBPI_{{M_{3} }} } \right) = 0.631$$,

$$S\left( {OBPI_{{M_{4} }} ,OBPI_{{M_{5} }} } \right) = S\left( {OBPI_{{M_{5} }} ,OBPI_{{M_{4} }} } \right) = 0.589$$, $$S\left( {OBPI_{{M_{4} }} ,OBPI_{{M_{6} }} } \right) = S\left( {OBPI_{{M_{6} }} ,OBPI_{{M_{4} }} } \right) = 0.688$$,

$$S\left( {OBPI_{{M_{4} }} ,OBPI_{{M_{7} }} } \right) = S\left( {OBPI_{{M_{7} }} ,OBPI_{{M_{4} }} } \right) = 0.721$$, $$S\left( {OBPI_{{M_{4} }} ,OBPI_{{M_{8} }} } \right) = S\left( {OBPI_{{M_{8} }} ,OBPI_{{M_{4} }} } \right) = 0.813$$,

$$S\left( {OBPI_{{M_{5} }} ,OBPI_{{M_{6} }} } \right) = S\left( {OBPI_{{M_{6} }} ,OBPI_{{M_{5} }} } \right) = 0.643$$, $$S\left( {OBPI_{{M_{5} }} ,OBPI_{{M_{7} }} } \right) = S\left( {OBPI_{{M_{7} }} ,OBPI_{{M_{5} }} } \right) = 0.717$$,

$$S\left( {OBPI_{{M_{5} }} ,OBPI_{{M_{8} }} } \right) = S\left( {OBPI_{{M_{8} }} ,OBPI_{{M_{5} }} } \right) = 0.589$$, $$S\left( {OBPI_{{M_{6} }} ,OBPI_{{M_{7} }} } \right) = S\left( {OBPI_{{M_{7} }} ,OBPI_{{M_{6} }} } \right) = 0.775$$,

$$S\left( {OBPI_{{M_{6} }} ,OBPI_{{M_{8} }} } \right) = S\left( {OBPI_{{M_{8} }} ,OBPI_{{M_{6} }} } \right) = 0.698$$, $$S\left( {OBPI_{{M_{7} }} ,OBPI_{{M_{8} }} } \right) = S\left( {OBPI_{{M_{8} }} ,OBPI_{{M_{7} }} } \right) = 0.656$$ .

Step 2: Similarity matrix is calculated as follow:

$$ \left[ {\begin{array}{*{20}c} 1 & {0.738} & {0.712} & {0.557} & {0.870} & {0.696} & {0.728} & {0.534} \\ {} & 1 & {0.794} & {0.567} & {0.676} & {0.879} & {0.791} & {0.593} \\ {} & {} & 1 & {0.721} & {0.693} & {0.755} & {0.906} & {0.631} \\ {} & {} & {} & 1 & {0.589} & {0.668} & {0.721} & {0.813} \\ {} & {} & {} & {} & 1 & {0.643} & {0.717} & {0.589} \\ {} & {} & {} & {} & {} & 1 & {0.775} & {0.698} \\ {} & {} & {} & {} & {} & {} & 1 & {0.656} \\ {} & {} & {} & {} & {} & {} & {} & 1 \\ \end{array} } \right] $$

Step 3: Based on step 2 and $$\zeta_{ij}^{\tau } = \,\left\{ \begin{gathered} 1,\,\,\,\,S\left( {OBPI_{{M_{i} }} ,OBPI_{{M_{j} }} } \right)\,\,\, \ge \,\,\tau \hfill \\ 0,\,\,\,S\left( {OBPI_{{M_{i} }} ,OBPI_{{M_{j} }} } \right)\,\,\, < \,\,\tau \hfill \\ \end{gathered} \right.\,\,,$$ the $\tau$-cutting matrix $D_{\tau } = \left( {\zeta_{ij}^{\tau } } \right)_{ij}$ can be developed.

$$\begin{gathered} \,\,\,\,\,\,\,\,\,\,\,(1)\,\,\,\,\,\,\,\,\,\,\,\,0.906 < \tau \le 1 \hfill \\ \,D_{\tau } = \left[ {\begin{array}{*{20}c} 1 & {} & {} & {} & {} & {} & {} & {} \\ {} & 1 & {} & {} & {} & {} & {} & {} \\ {} & {} & 1 & {} & {} & {} & {} & {} \\ {} & {} & {} & 1 & {} & {} & {} & {} \\ {} & {} & {} & {} & 1 & {} & {} & {} \\ {} & {} & {} & {} & {} & 1 & {} & {} \\ {} & {} & {} & {} & {} & {} & 1 & {} \\ {} & {} & {} & {} & {} & {} & {} & 1 \\ \end{array} } \right] \hfill \\ \end{gathered}$$ $$\begin{gathered} \,\,\,\,\,\,\,\,\,\,\,(2)\,\,\,\,\,\,\,\,0.879 < \tau \le 0.906 \hfill \\ \,D_{\tau } = \left[ {\begin{array}{*{20}c} 1 & {} & {} & {} & {} & {} & {} & {} \\ {} & 1 & {} & {} & {} & {} & {} & {} \\ {} & {} & 1 & {} & {} & {} & 1 & {} \\ {} & {} & {} & 1 & {} & {} & {} & {} \\ {} & {} & {} & {} & 1 & {} & {} & {} \\ {} & {} & {} & {} & {} & 1 & {} & {} \\ {} & {} & {} & {} & {} & {} & 1 & {} \\ {} & {} & {} & {} & {} & {} & {} & 1 \\ \end{array} } \right] \hfill \\ \end{gathered}$$

$$\begin{gathered} \,\,\,\,\,\,\,\,\,\,\,(3)\,\,\,\,\,\,\,\,\,\,0.870 < \tau \le 0.879 \hfill \\ \,D_{\tau } = \left[ {\begin{array}{*{20}c} 1 & {} & {} & {} & {} & {} & {} & {} \\ {} & 1 & {} & {} & {} & 1 & {} & {} \\ {} & {} & 1 & {} & {} & {} & 1 & {} \\ {} & {} & {} & 1 & {} & {} & {} & {} \\ {} & {} & {} & {} & 1 & {} & {} & {} \\ {} & {} & {} & {} & {} & 1 & {} & {} \\ {} & {} & {} & {} & {} & {} & 1 & {} \\ {} & {} & {} & {} & {} & {} & {} & 1 \\ \end{array} } \right] \hfill \\ \end{gathered}$$ $$\begin{gathered} \,\,\,\,\,\,\,\,\,\,\,(4)\,\,\,\,\,\,\,\,\,\,0.813 < \tau \le 0.870 \hfill \\ \,D_{\tau } = \left[ {\begin{array}{*{20}c} 1 & {} & {} & {} & 1 & {} & {} & {} \\ {} & 1 & {} & {} & {} & 1 & {} & {} \\ {} & {} & 1 & {} & {} & {} & 1 & {} \\ {} & {} & {} & 1 & {} & {} & {} & {} \\ {} & {} & {} & {} & 1 & {} & {} & {} \\ {} & {} & {} & {} & {} & 1 & {} & {} \\ {} & {} & {} & {} & {} & {} & 1 & {} \\ {} & {} & {} & {} & {} & {} & {} & 1 \\ \end{array} } \right] \hfill \\ \end{gathered}$$

$$\begin{gathered} \,\,\,\,\,\,\,\,\,\,\,(5)\,\,\,\,\,\,\,0.794 < \tau \le 0.813 \hfill \\ \,D_{\tau } = \left[ {\begin{array}{*{20}c} 1 & {} & {} & {} & 1 & {} & {} & {} \\ {} & 1 & {} & {} & {} & 1 & {} & {} \\ {} & {} & 1 & {} & {} & {} & 1 & {} \\ {} & {} & {} & 1 & {} & {} & {} & 1 \\ {} & {} & {} & {} & 1 & {} & {} & {} \\ {} & {} & {} & {} & {} & 1 & {} & {} \\ {} & {} & {} & {} & {} & {} & 1 & {} \\ {} & {} & {} & {} & {} & {} & {} & 1 \\ \end{array} } \right] \hfill \\ \end{gathered}$$ $$\begin{gathered} \,\,\,\,\,\,\,\,\,\,\,(6)\,\,\,\,\,\,\,\,0.791 < \tau \le 0.794 \hfill \\ \,D_{\tau } = \left[ {\begin{array}{*{20}c} 1 & {} & {} & {} & 1 & {} & {} & {} \\ {} & 1 & 1 & {} & {} & 1 & {} & {} \\ {} & {} & 1 & {} & {} & {} & 1 & {} \\ {} & {} & {} & 1 & {} & {} & {} & 1 \\ {} & {} & {} & {} & 1 & {} & {} & {} \\ {} & {} & {} & {} & {} & 1 & {} & {} \\ {} & {} & {} & {} & {} & {} & 1 & {} \\ {} & {} & {} & {} & {} & {} & {} & 1 \\ \end{array} } \right] \hfill \\ \end{gathered}$$$$\begin{gathered} \,\,\,\,\,\,\,\,\,\,\,(7)\,\,\,\,\,\,\,0.775 < \tau \le 0.791 \hfill \\ \,D_{\tau } = \left[ {\begin{array}{*{20}c} 1 & {} & {} & {} & 1 & {} & {} & {} \\ {} & 1 & 1 & {} & {} & 1 & 1 & {} \\ {} & {} & 1 & {} & {} & {} & 1 & {} \\ {} & {} & {} & 1 & {} & {} & {} & 1 \\ {} & {} & {} & {} & 1 & {} & {} & {} \\ {} & {} & {} & {} & {} & 1 & {} & {} \\ {} & {} & {} & {} & {} & {} & 1 & {} \\ {} & {} & {} & {} & {} & {} & {} & 1 \\ \end{array} } \right] \hfill \\ \end{gathered}$$ $$\begin{gathered} \,\,\,\,\,\,\,\,\,\,\,(8)\,\,\,\,\,\,\,\,0.738 < \tau \le 0.755 \hfill \\ \,D_{\tau } = \left[ {\begin{array}{*{20}c} 1 & {} & {} & {} & 1 & {} & {} & {} \\ {} & 1 & 1 & {} & {} & 1 & 1 & {} \\ {} & {} & 1 & {} & {} & {} & 1 & {} \\ {} & {} & {} & 1 & {} & {} & {} & 1 \\ {} & {} & {} & {} & 1 & {} & {} & {} \\ {} & {} & {} & {} & {} & 1 & 1 & {} \\ {} & {} & {} & {} & {} & {} & 1 & {} \\ {} & {} & {} & {} & {} & {} & {} & 1 \\ \end{array} } \right] \hfill \\ \end{gathered}$$

$$\begin{gathered} \,\,\,\,\,\,\,\,\,\,\,(9)\,\,\,\,\,\,\,\,\,\,0 < \tau \le 0.721 \hfill \\ \,D_{\tau } = \left[ {\begin{array}{*{20}c} 1 & {} & {} & {} & 1 & {} & {} & {} \\ {} & 1 & 1 & {} & {} & 1 & 1 & {} \\ {} & {} & 1 & {} & {} & 1 & 1 & {} \\ {} & {} & {} & 1 & {} & {} & {} & 1 \\ {} & {} & {} & {} & 1 & {} & {} & {} \\ {} & {} & {} & {} & {} & 1 & 1 & {} \\ {} & {} & {} & {} & {} & {} & 1 & {} \\ {} & {} & {} & {} & {} & {} & {} & 1 \\ \end{array} } \right] \hfill \\ \end{gathered}$$

If $0.906 < \tau \le 1$, then $M$ is classified in to these eight types;

$$ \left\{ {M_{1} } \right\},\,\,\left\{ {M_{2} } \right\},\,\,\left\{ {M_{3} } \right\},\,\,\left\{ {M_{4} } \right\},\,\,\left\{ {M_{5} } \right\},\,\,\left\{ {M_{6} } \right\},\,\,\left\{ {M_{7} } \right\},\left\{ {M_{8} } \right\}. $$

If $0.879 < \tau \le 0.906$, then $M$ is classified into these seven types;

$$\left\{ {M_{1} } \right\},\left\{ {M_{2} } \right\},\left\{ {M_{3} ,M_{7} } \right\},\left\{ {M_{4} } \right\},\left\{ {M_{5} } \right\},\left\{ {M_{6} } \right\},\left\{ {M_{8} } \right\}$$.

If $0.870 < \tau \le 0.879$ then $M$ is classified into these six types;

$$\left\{ {M_{1} } \right\},\left\{ {M_{2} ,M_{6} } \right\},\left\{ {M_{3} ,M_{7} } \right\},\left\{ {M_{4} } \right\},\left\{ {M_{5} } \right\},\left\{ {M_{8} } \right\}$$.

If $0.813 < \tau \le 0.870$ then $M$ is classified into these five types;

$$ \left\{ {M_{1} ,M_{5} } \right\},\left\{ {M_{2} ,M_{6} } \right\},\left\{ {M_{3} ,M_{7} } \right\},\left\{ {M_{4} } \right\},\left\{ {M_{8} } \right\}. $$

If $0.794 < \tau \le 0.813$ then, $M$ is classified into these four types;

$$ \left\{ {M_{1} ,M_{5} } \right\},\left\{ {M_{2} ,M_{6} } \right\},\left\{ {M_{3} \,,M_{7} } \right\},\left\{ {M_{4} \,,M_{8} } \right\}. $$

If $0.791 < \tau \le 0.794$ then, $M$ is classified into these three types;

$$ \left\{ {M_{1} ,M_{5} } \right\},\left\{ {M_{2} ,M_{3} ,M_{6} ,M_{7} } \right\},\left\{ {M_{4} ,M_{8} } \right\}. $$

If $0.738 < \tau \le 0.755$ then, $M$ is classified into these two types;

$$ \left\{ {M_{1} ,M_{2} ,M_{3} ,M_{5} ,M_{6} ,M_{7} } \right\},\left\{ {M_{4} ,M_{8} } \right\}. $$

If $0 < \tau \le 0.721$ then, $M$ are of same type:

$$ \left\{ {M_{1} ,\,\,M_{2} ,\,M_{3} ,\,\,M_{4} ,\,\,M_{5} ,\,\,M_{6} ,\,\,M_{7} ,M_{8} } \right\}. $$

**Figure 3:**
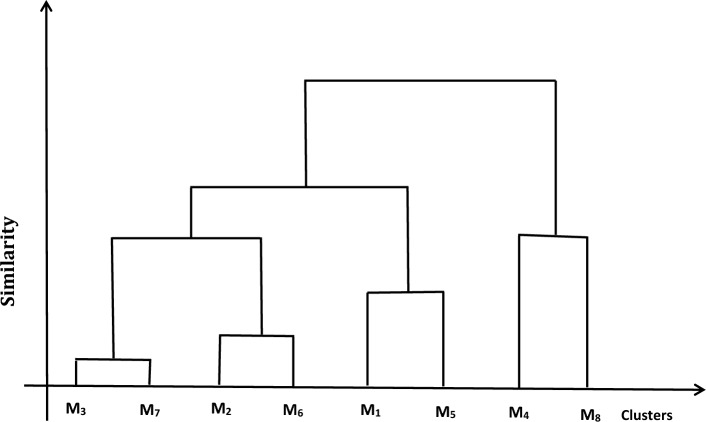
Dendrogram for the hierarchical clustering.

When the function $f$ takes different forms, the resulting clusters are unchanged, despite the ranges of confidence level may be vary. So from the real clustering processes, the decision-makers can choose the suitable function $f$ regarding the actual situations. Figure 3 shows the dendrogram for agglomerative hierarchical clustering.

The above example shows the applicability and validity of our proposed similarity measure Eq. (10) between two q-ROFSs in clustering. According to the different $\tau$ levels, we may use our similarity measure Eq. (10) between two q-ROFSs to construct a hierarchical clustering for the data in the form of q-ROFSs.

### Comparison of proposed measures with existing ones

We compare our proposed measures with the recently suggested distance measure between q-ROFSs given by Wang et al.^24^ to show the superiority, suitability and reasonability of our proposed measures. The distance measure between q-ROFSs given by Wang et al.^24^ is as follows:

13 $$ \begin{gathered} D_{JS\_2D} \left( {Q_{1} ,Q_{2} } \right) = \left[ {\frac{1}{2}\left( {\chi_{{Q_{1} }}^{q} \left( x \right)Log\frac{{2\chi_{{Q_{1} }}^{q} \left( x \right)}}{{\chi_{{Q_{1} }}^{q} \left( x \right) + \chi_{{Q_{2} }}^{q} \left( x \right)}} + \chi_{{Q_{2} }}^{q} \left( x \right)Log\frac{{2\chi_{{Q_{2} }}^{q} \left( x \right)}}{{\chi_{{Q_{1} }}^{q} \left( x \right) + \chi_{{Q_{2} }}^{q} \left( x \right)}}} \right.} \right. \hfill \\ \left. {\left. {\,\,\,\,\,\,\,\,\,\,\,\,\,\,\,\,\,\,\,\,\,\,\,\,\,\,\,\,\,\,\,\,\,\,\,\,\,\,\,\,\,\,\,\,\,\,\, + \vartheta_{{Q_{1} }}^{q} \left( x \right)Log\frac{{2\vartheta_{{Q_{1} }}^{q} \left( x \right)}}{{\vartheta_{{Q_{1} }}^{q} \left( x \right) + \vartheta_{{Q_{2} }}^{q} \left( x \right)}} + \vartheta_{{Q_{2} }}^{q} \left( x \right)Log\frac{{2\vartheta_{{Q_{2} }}^{q} \left( x \right)}}{{\vartheta_{{Q_{1} }}^{q} \left( x \right) + \vartheta_{{Q_{2} }}^{q} \left( x \right)}}} \right)} \right]^{\frac{1}{2}} \hfill \\ \end{gathered} $$

For comparison, we extract similarity measure from the above distance by using the relation ${\text{similarity}}\,{ = 1 - }\,{\text{distance}}$ as:

14 $$ S\left( {Q_{1} ,Q_{2} } \right) = 1 - D_{JS\_2D} \left( {Q_{1} ,Q_{2} } \right) $$

We use the example 3 of manuscript in which three different patterns are given which are expressed by three q-ROFSs $M_{1}$, $M_{2}$ and $M_{3}$ respectively, and can be represented as:

$M_{1} = \left\{ {\left\langle {z_{1} ,0.4,0.8} \right\rangle ,\left\langle {z_{2} ,0.5,0.7} \right\rangle ,\left\langle {z_{3} ,0.6,0.5} \right\rangle } \right\}$, $M_{2} = \left\{ {\left\langle {z_{1} ,0.2,0.9} \right\rangle ,\left\langle {z_{2} ,0.4,0.7} \right\rangle ,\left\langle {z_{3} ,0.6,0.7} \right\rangle } \right\}$ and $M_{3} = \left\{ {\left\langle {z_{1} ,0.7,0.6} \right\rangle ,\left\langle {z_{2} ,0.9,0.8} \right\rangle ,\left\langle {z_{3} ,0.8,0.6} \right\rangle } \right\}$ on the universe of discourse $Z = \left\{ {z_{1} ,z_{2} ,z_{3} } \right\}$**.**

Another pattern $N = \left\{ {\left\langle {z_{1} ,0.5,0.7} \right\rangle ,\left\langle {z_{2} ,0.5,0.6} \right\rangle ,\left\langle {z_{3} ,0.7,0.5} \right\rangle } \right\}$ is given as a sample. Our aim is to identify the pattern to which the sample $N$ resembles. The results of the proposed similarity measures Eqs. (8)–(10) are:

$$S_{L} \left( {OBPI_{{M_{1} }} ,OBPI_{N} } \right) = 0.859,$$ $$S_{L} \left( {OBPI_{{M_{2} }} ,OBPI_{N} } \right) = 0.756,$$ $$S_{L} \left( {OBPI_{{M_{3} }} ,OBPI_{N} } \right) = 0.670,$$

$$S_{Q} \left( {OBPI_{{M_{1} }} ,OBPI_{N} } \right) = 0.753,$$ $$S_{Q} \left( {OBPI_{{M_{2} }} ,OBPI_{N} } \right) = 0.608,$$ $$S_{Q} \left( {OBPI_{{M_{3} }} ,OBPI_{N} } \right) = 0.401,$$

$$S_{E} \left( {OBPI_{{M_{1} }} ,OBPI_{N} } \right) = 0.792,$$ $$S_{E} \left( {OBPI_{{M_{2} }} ,OBPI_{N} } \right) = 0.657,$$ $$S_{E} \left( {OBPI_{{M_{3} }} ,OBPI_{N} } \right) = 0.555.$$

The results obtained from Eq. (14) are:

$$S\left( {M_{1} ,N} \right) = 0.9364$$, $$S\left( {M_{2} ,N} \right) = 0.8724$$, $$S\left( {M_{3} ,N} \right) = 0.8310$$.

The above results show that the given sample $N$ resembles with the pattern $M_{1}$ which confirms the correctness of our proposed measures. However, the existing distance measure of Eq. (13) has some draw backs. For example, if we consider degenerated case of IFS $M_{i} = \left\{ {\left\langle {z,1,0} \right\rangle } \right\}$ or $M_{i} = \left\{ {\left\langle {z,0,1} \right\rangle } \right\}$ then, the existing distance measure of Eq. (13) become undefined. In contrast, our proposed measures handle this situation amicably. This effectively demonstrates the superiority of our proposed divergence measure over the existing one.

## Construction of Orthopairian belief and plausible GRA

Making decisions is a regular activity that we all engage in on a daily basis. When using multiple criteria, we choose the best option by ranking all of the available options. To achieve this, it is important to ensure a better decision-making process.

GRA was first developed by Deng^55^ to handle imprecise and incomplete information in MCDM in grey setting. Literature shows that GRA has been employed by many researchers in q-ROFSs environment^56,57^ to find the best option from the given alternatives on the basis of respective Relative Grey Relational Degree (RGRD). In this section, we discuss our work on distance measure between belief and plausibility of q-ROFS which helps us provide a good way to tackle the MCCDM. We extended GRA technique to develop belief and plausible GRA with belief-plausible intervals of q-ROFS to solve multicriteria decision making issues.

We proposed a novel technique to tackle MCCDM issues with unidentified weights. The suggested distance measure among OBPIs is used to measure the distance between the alternatives $A_{i} \,$ from belief-plausibility negative ideal solutions (BP-NIS) and belief-plausibility positive ideal solutions (BP-PIS) respectively and then utilization of GRA process for ranking of alternatives $A_{i} \,\left\{ {i = 1,2,...,m} \right\}$ and criterias $C_{j} \left\{ {j = 1,2,...,n} \right\}$. The purpose of this problem is to select the best alternative among all existing alternatives. The step-wise algorithm of belief-Plausibility GRA is under.

Step 1: Construction of belief-plausibility decision matrix.

Let us consider $A = \left\{ {A_{1} ,A_{2} ,...,A_{m} } \right\}$ be a set of alternatives to criteria $C = \left\{ {C_{1} ,C_{2} ,...,C_{n} } \right\}$. The decision making matrix for q-ROFSs be $\tilde{D} = \left[ {\tilde{b}_{ij} } \right]_{m \times n} = \left[ {Bl_{ij} ,\,Pl_{ij} } \right]$, where $Bl_{ij} = \mu_{ij}^{q}$ and $Pl_{ij} = 1 - \nu_{ij}^{q} .$ The value of $Bl_{ij} = \mu_{ij}^{q}$ indicates the degree of belief and $Pl_{ij} = 1 - \nu_{ij}^{q}$ indicates the plausible degree against the alternatives $A_{i}$ to criteria $C_{j}$ in such a way that $0 \le Bl_{ij} \le 1\,\,\,,\,\,\,0 \le Pl_{ij} \le 1\,$ and $0 \le Bl_{ij} \le Pl_{ij} \le 1$ remains unchanged. The decision matrix will be established as follows:

$$ \tilde{D} = \left[ {\widehat{{h_{ij} }}} \right]_{m \times n} = \left[ {\begin{array}{*{20}c} {\left[ {Bl_{11} ,Pl_{11} } \right]} & {\left[ {Bl_{12} ,Pl_{12} } \right]} & . & . & . & {\left[ {Bl_{1n} ,Pl_{1n} } \right]} \\ {\left[ {Bl_{21} ,Pl_{21} } \right]} & {\left[ {Bl_{22} ,Pl_{22} } \right]} & . & . & . & {\left[ {Bl_{2n} ,Pl_{2n} } \right]} \\ . & . & . & . & . & . \\ . & . & . & . & . & . \\ . & . & . & . & . & . \\ {\left[ {Bl_{m1} ,Pl_{m1} } \right]} & {\left[ {Bl_{m2} ,Pl_{m2} } \right]} & . & . & . & {\left[ {Bl_{mn} ,Pl_{mn} } \right]} \\ \end{array} } \right] $$

Step 2: Normalize the decision matrix.

$$ \tilde{D}_{ } = \left\{ {\begin{array}{*{20}c} {I_{ij} = \left( {\mu_{ij} , v_{ij} } \right), } & {if C_{j} \in Cb (benefit \,\,criteria)} \\ {( I_{ij} )^{c} = \left( {v_{ij} , \mu_{ij} } \right),} & {if C_{j} \in Cc ({\text{Cos}} t \,\,criteria)} \\ \end{array} } \right. $$

Step 3: Determination of the weight of criterion.

Here, we find the weights of criterion. To find the weight criterias there are many ways. Let us consider that the weights of criterias $$C_{j} \left\{ {j = 1,2,...,n} \right\}\,$$ are $$\omega_{j} ,\,j = 1,2,...,n$$ with $$\sum\limits_{j = 1}^{n} {\omega_{j} = 1}$$ and also $$\,0 \le \omega_{j} \le 1$$. Here weights are not given, so we suggest a new way to find weights as: $$\omega_{j} = \frac{{\left( {{\raise0.7ex\hbox{${\left( {3Bl_{j} + Pl_{j} } \right)}$} \!\mathord{\left/ {\vphantom {{\left( {3Bl_{j} + Pl_{j} } \right)} 2}}\right.\kern-0pt} \!\lower0.7ex\hbox{$2$}}} \right)}}{{\sum\limits_{i = 1}^{n} {\left( {{\raise0.7ex\hbox{${\left( {3Bl_{j} + Pl_{j} } \right)}$} \!\mathord{\left/ {\vphantom {{\left( {3Bl_{j} + Pl_{j} } \right)} 2}}\right.\kern-0pt} \!\lower0.7ex\hbox{$2$}}} \right)} }}$$.

Step 4: Positive and negative ideal solutions.

In this step we divide the criteria into two parts: one is for beneficial criteria and the other is for cost criteria denoted by $B_{1}$ and $B_{2}$ respectively, which satisfying the property $B_{1} \subseteq C\,\,\,{\text{and}}\,\,\,B_{2} \subseteq C$ and $B_{1} \cap B_{2} = \phi .$ By the principle of GRA in belief and plausible q-ROF positive ideal solution (BP-PIS) and belief and plausible negative ideal solution (BP-NIS) is given below:

$BP - PIS \, = \, I^{ + } = \left( {Bl_{ij}^{ + } , \, Pl_{ij}^{ + } } \right) \, = \left( {\mathop {\max }\limits_{i} Bl_{ij} ,\mathop {\min }\limits_{i} Pl_{ij} } \right)$ for higher the best and

$BP - NIS \, = \, I^{ - } = \left( {Bl_{ij}^{ - } , \, Pl_{ij}^{ - } } \right) \, = \left( {\mathop {\min }\limits_{i} Bl_{ij} ,\mathop {\max }\limits_{i} Pl_{ij} } \right)$ for lower the best.

Step 5: Calculate the q-ROF distance matrices:

(i) Calculate the q-ROF-distance by using proposed distance between (BP-PIS) and q-ROF value of each alternative: $D^{ + } = \, \left[ {D\left( {I_{ij} ,\,I_{j}^{ + } } \right)} \right]_{m \, \times \, n}$
(ii) Evaluate the q-ROF-distance between (BP-NIS) and q-ROF value of each alternative: $D^{ - } = \, \left[ {D\left( {I_{ij} ,\,I_{j}^{ - } } \right)} \right]_{m \, \times \, n}$

Step 6: Find the grey relational coefficient (GRC) Matrix:

(a) Calculate the GRC of each alternative from BP-PIS:

$$ {\upxi }_{ij}^{ + } = \,\,\frac{{\mathop {\min }\limits_{1\, \le \,\,i\,\, \le \,m} \,\,\mathop {\min }\limits_{1\, \le \,j\, \le \,\,n} \,\,D(I_{ij} ,\,I_{j}^{ + } )\, + \,\rho .\,\mathop {\max }\limits_{1\, \le \,\,i\,\, \le \,m} \,\,\mathop {\max }\limits_{1\, \le \,j\, \le \,n} \,D(I_{ij} ,\,I_{j}^{ + } )}}{{\,\,D(I_{ij} ,\,I_{j}^{ + } )\, + \,\rho .\,\mathop {\max }\limits_{1\,\, \le \,\,i\,\, \le \,\,m} \,\,\mathop {\max }\limits_{1\,\, \le j\, \le \,n} \,D(I_{ij} ,\,I_{j}^{ + } )}}. $$

(b) Calculate the GRC of each alternative from BP-NIS:

$$ {\upxi }_{ij}^{ - } = \,\,\frac{{\mathop {\min }\limits_{1\,\, \le \,\,i\,\, \le \,\,m} \,\,\mathop {\min }\limits_{1\,\, \le j\,\, \le \,\,n} \,\,D(I_{ij} ,\,I_{j}^{ - } )\, + \,\rho .\,\mathop {\max }\limits_{1\,\, \le \,\,i\,\, \le \,\,m} \,\,\mathop {\max }\limits_{1\, \le \,\,j\,\, \le \,\,n} \,D(I_{ij} ,\,I_{j}^{ - } )}}{{\,\,D(I_{ij} ,\,I_{j}^{ - } )\, + \,\rho .\,\mathop {\max }\limits_{1\,\, \le \,i\, \le \,m} \,\,\mathop {\max }\limits_{1\,\, \le \,\,j\,\, \le \,\,n} \,D(I_{ij} ,\,I_{j}^{ - } )}}. $$

where $\rho$ is identification coefficient and taken $\rho$ =0.5 for stabilization.

Step 7: Evaluate weighted grey relational degrees (GRD):

We use the above equations of (a) and (b) to find weighted grey relational degrees ${\upxi }_{j}^{ + }$ and ${\upxi }_{j}^{ - }$, respectively.

(i) $$\xi_{j}^{ + } = \sum\limits_{j = 1}^{n} {\omega_{j} . I_{ij}^{ + } }$$ (ii) $$\xi_{j}^{ - } = \sum\limits_{j = 1}^{n} {\omega_{j} . I_{ij}^{ - } }$$.

Step 8: Find relative grey relational degree RGRD for each alternative:

Calculate relative grey relational degree using the below formula:

$$ \,\xi_{i} = \frac{{\xi_{j}^{ + } }}{{\xi_{j}^{ + } + \xi_{j}^{ - } }} $$

Step 9: Ranking:

Finally, the alternatives are ranked on the basis of their increasing RGRD values.

**Figure 4:**
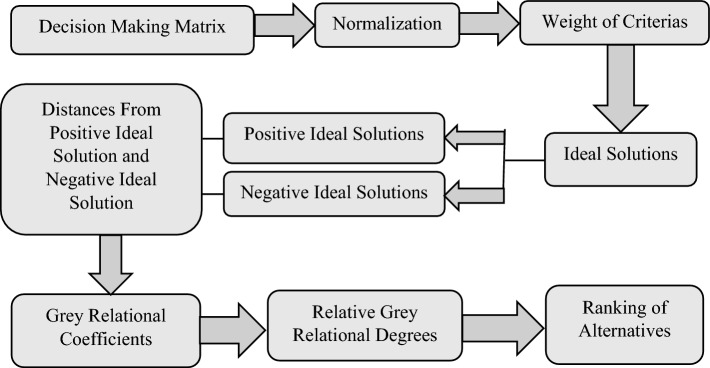
Flow chart for the new modified OBP-GRA algorithm.

The flow chart of our proposed OBP-GRA algorithm is shown in Fig. 4 below.

Now, the above algorithm is used to tackle a daily life complex problem.

### Example 4

COVID-19 sickness caused by coronavirus SARS-CoV-2 is the most alarming and disastrous issue of almost all over the world since 11th March 2020 which is rapidly increasing day by day and is declared as pandemic by World Health Organization (WHO)^58^. More than 72 countries are affected with this deadly virus with almost 772 million confirmed cases and more than 6 million death cases so far which is updated on 19th November, 2023^59^. According to the Covid-19 epidemiological update of 19th January, 2024, during the 28-day period from December 11, 2023, to January 7, 2024, nearly 1.1 million new cases were reported worldwide, a 4% increase over the previous 28-day period. With 8700 new deaths recorded, there were 26% fewer new deaths than there were during the preceding 28 days. As of January 7, 2024, there had been over seven million recorded deaths and over 774 million confirmed cases worldwide^60,61^.

Numerous COVID-19 vaccines have been approved for use by WHO. Early in December 2020, the first mass vaccination campaign was launched. There are several vaccines introduced to overcome the variants of this deadly pandemic. Some vaccines are too costly and not affordable for under-developed countries, and some are easily available. Some of the vaccines are rapidly effective but have number of side effects. So it is much more difficult for a common man affected by COVID-19 to choose the most effective vaccine among all. To check the effectiveness of these vaccines, assume that there are four possible alternatives: (a) Vaccine $A_{1}$, (b) Vaccine $A_{2}$, (c) Vaccine $A_{3}$ and (d) Vaccine $A_{4}$. Based upon the viewpoint of the relevant medical experts, the following four important criterias are set to assess these four vaccines. These criterias are: $C_{1}$ = Easy Access and Availability, $C_{2}$ = Effectiveness, $C_{3}$ = Long-term immunity, and $C_{4}$ = Side effects. A thorough summary of the criterias are mentioned in Table 1. The evaluations of alternatives $A_{i}$ to the criterias $C_{j}$ are shown in Table 2 in terms of belief and plausible decision making matrix $\tilde{D} = \left( {A_{i} \left( {C_{j} } \right)} \right)_{m \times n}$. The characteristic sets of alternative $A_{i}$ are as follows:

$$ A_{1} = \left\{ {\left\langle {z_{1} ,0.8,0.6} \right\rangle ,\left\langle {z_{2} ,0.5,0.7} \right\rangle ,\left\langle {z_{3} ,0.6,0.7} \right\rangle ,\left\langle {z_{4} ,0.7,0.6} \right\rangle } \right\} $$

$$ A_{2} = \left\{ {\left\langle {z_{1} ,0.9,0.5} \right\rangle ,\left\langle {z_{2} ,0.7,0.9} \right\rangle ,\left\langle {z_{3} ,1.0,0.0} \right\rangle ,\left\langle {z_{4} ,0.6,0.7} \right\rangle } \right\} $$

$$ A_{3} = \left\{ {\left\langle {z_{1} ,0.9,0.9} \right\rangle ,\left\langle {z_{2} ,0.8,0.5} \right\rangle ,\left\langle {z_{3} ,0.7,0.7} \right\rangle ,\left\langle {z_{4} ,0.8,0.6} \right\rangle } \right\} $$

$$ A_{4} = \left\{ {\left\langle {z_{1} ,0.7,0.6} \right\rangle ,\left\langle {z_{2} ,0.9,0.5} \right\rangle ,\left\langle {z_{3} ,0.8,0.8} \right\rangle ,\left\langle {z_{4} ,0.8,0.6} \right\rangle } \right\} $$

**Table 1:** Description of criteria.

Criterion	Description of criterion
Easy access and availability, C_1_	The vaccine should be easily available and accessible for everyone according to their needs
Effectiveness, C_2_	The vaccine should be effective against the disease rapidly and cure the patients early
Long-term immunity, C_3_	The vaccine should have long-term immunity against the disease
Side effects, C_4_	The vaccine should be free of any side effect after usage, like itching of skin, dizziness, headache, high blood pressure etc

**Table 2:** Belief and plausible decision matrix.

Alternatives/criterion	*C*_1_	*C*_2_	*C*_3_	*C*_4_
*A*_1_	[0.512, 0.784]	[0.125, 0.657]	[0.216, 0.657]	[0.343, 0.784]
*A*_2_	[0.729, 0.875]	[0.343, 0.271]	[1.000, 1.000]	[0.216, 0.657]
*A*_3_	[0.729, 0.271]	[0.512, 0.875]	[0.343, 0.657]	[0.512, 0.784]
*A*_4_	[0.343, 0.784]	[0.729, 0.875]	[0.512, 0.488]	[0.512, 0.784]

Table 1 consists of the detailed summary of the criterias.

Step 1 Constructing belief and plausible decision matrix.

$$\overset{\lower0.5em\hbox{$\smash{\scriptscriptstyle\frown}$}}{A} = \left[ {\widehat{h}_{ih} } \right]_{m \times n} = \left[ {Bl_{ij} ,Pl_{ij} } \right]$$ with their belief and plausible decision matrix $$A^{ * } = \left[ {h^{ * }_{ih} } \right]_{m \times n} = \left[ {Bl_{ij} ,Pl_{ij} } \right]\,\,\,$$ where $$Bl_{ij} = \mu_{ij}^{q}$$ and $$Pl_{ij} = 1 - \nu_{ij}^{q}$$, explicitly the alternatives $$A = \left\{ {A_{1} ,A_{2} ,...,A_{m} } \right\}$$ by the distinctive set $$A_{i} = \left\{ {\left\langle {C_{j} ,\left[ {Bl_{ij} ,Pl_{ij} } \right]} \right\rangle :C_{j} \in C} \right\}$$.

As, cost criteria is not involved, so there is no need of step 2.

Step 3 Determining the weights of criterion.

The weight for each criteria is computed from proposed weighting formula $\omega_{j} = {{\left( {{{\left( {3Bl_{j} + Pl_{j} } \right)} \mathord{\left/ {\vphantom {{\left( {3Bl_{j} + Pl_{j} } \right)} 2}} \right. \kern-0pt} 2}} \right)} \mathord{\left/ {\vphantom {{\left( {{{\left( {3Bl_{j} + Pl_{j} } \right)} \mathord{\left/ {\vphantom {{\left( {3Bl_{j} + Pl_{j} } \right)} 2}} \right. \kern-0pt} 2}} \right)} {\sum\limits_{i = 1}^{n} {\left( {{{\left( {3Bl_{j} + Pl_{j} } \right)} \mathord{\left/ {\vphantom {{\left( {3Bl_{j} + Pl_{j} } \right)} 2}} \right. \kern-0pt} 2}} \right)} }}} \right. \kern-0pt} {\sum\limits_{i = 1}^{n} {\left( {{{\left( {3Bl_{j} + Pl_{j} } \right)} \mathord{\left/ {\vphantom {{\left( {3Bl_{j} + Pl_{j} } \right)} 2}} \right. \kern-0pt} 2}} \right)} }}$. The calculated weights for each criterion are:

$$ \omega_{1} = 0.2403,\,\,\,\omega_{2} = 0.3172,\,\,\omega_{3} = 0.2546,\,\,\,\omega_{4} = 0.1878 $$

Step 4 Constructing belief and plausible ideal solutions.

The BP-PIS and BP-NIS by considering the cost and benefit criterion are as under.

$$\,I^{ + } = \left\{ {\left\langle {z_{1} ,0,729,0.271} \right\rangle ,\left\langle {z_{2} ,0.729,0.271} \right\rangle ,\left\langle {z_{3} ,1.000,0.488} \right\rangle ,\left\langle {z_{4} ,0.512,0.657} \right\rangle } \right\}$$,$$\,I^{ - } = \left\{ {\left\langle {z_{1} ,0.343,0.875} \right\rangle ,\left\langle {z_{2} ,0.125,0.875} \right\rangle ,\left\langle {z_{3} ,0.216,1.000} \right\rangle ,\left\langle {z_{4} ,0.216,0.784} \right\rangle } \right\}$$.

Step 5: Calculating the distance matrix.

The distance matrix using Hausdorff distance formula between alternative $A_{i}$ to positive ideal solution $I^{ + }$ and negative ideal solution $I^{ - }$ is as follows:

$$D^{ + } = \left[ {\begin{array}{*{20}c} {0.513} & {0.604} & {0.784} & {0.169} \\ {0.604} & {0.386} & {0.512} & {0.296} \\ 0 & {0.604} & {0.657} & {0.127} \\ {0.513} & {0.604} & {0.488} & {0.127} \\ \end{array} } \right]$$, $$D^{ - } = \left[ {\begin{array}{*{20}c} {0.169} & {0.218} & {0.343} & {0.127} \\ {0.386} & {0.604} & {0.784} & {0.127} \\ {0.604} & {0.387} & {0.343} & {0.296} \\ {0.091} & {0.604} & {0.512} & {0.296} \\ \end{array} } \right]$$.

Step 6: The GRC matrix will be.

$$\xi^{ + } = \left[ {\begin{array}{*{20}c} {0.4332} & {0.5633} & {0.4770} & {0.9251} \\ {0.3333} & {0.8692} & {0.7611} & {0.7174} \\ 1 & {0.3523} & {0.3737} & {0.7212} \\ {0.3706} & {0.4735} & {0.5898} & 1 \\ \end{array} } \right]$$, $$\xi^{ - } = \left[ {\begin{array}{*{20}c} {0.8344} & {0.8250} & {0.7061} & 1 \\ {0.6671} & {0.5211} & {0.4413} & 1 \\ {0.4338} & {0.7547} & {0.9361} & {0.7174} \\ 1 & {0.4338} & {0.5343} & {0.6572} \\ \end{array} } \right]$$.

Step 7: The weighted grey relational degrees will be.

Table 3 represents the weighted grey relational degrees of alternatives.

**Table 3:** Weighted grey relational degrees.

ξ_1_^+^ = 0.5780	ξ_1_^–^ = 0.8298
ξ_2_^+^ = 0.6843	ξ_2_^–^ = 0.6258
ξ_3_^+^ = 0.5826	ξ_3_^–^ = 0.7167
ξ_4_^+^ = 0.5772	ξ_4_^–^ = 0.6374

Step 8: The relative grey relational degrees for each alternative will be calculated in the Table 4 as:

**Table 4:** Relative grey relational degrees.

ϒ_1_ = 0.4106
ϒ_2_ = 0.5223
ϒ_3_ = 0.4484
ϒ_4_ = 0.4752

Table 4 shows the relative grey relational degrees which are used to rank the alternatives.

Step 9: The final ranking of the alternatives according to the increasing values of relative grey relational degrees will be shown in Table 5.

**Table 5:** Ranking order of alternatives.

Ranking	Best alternative
*A*_2_ > *A*_4_ > *A*_3_ > *A*_1_	*A*_2_

**Figure 5:**
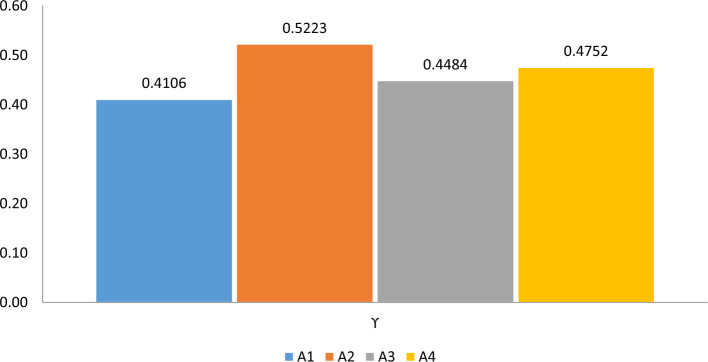
Ranking of alternatives on the basis of increasing RGRD values.

Table 5 clearly shows that the alternative $\left( {A_{2} } \right)$ is considered as the most suitable alternative. So for the effective treatment of deadly COVID-19, $\left( {A_{2} } \right)$ vaccine will be more effective than all other available vaccines which are also shown in Fig. 5 as below:

Figure 5 clearly shows that the alternative $\left( {A_{2} } \right)$ is the most suitable and best alternative among all other given alternatives.

## Conclusions

The belief and plausible functions in the framework of q-ROFSs allows for a more dynamic and context aware representation of vague, uncertain and incomplete information. Since, the literature contains no generalization of ET to q-ROFSs. Therefore, we have expressed belief in term of membership and plausibility in term of 1-nonmembership functions of q-ROFSs respectively which leads to generalization of ET to q-ROFSs. Then, the OBPIs based on suggested belief and plausible measures of q-ROFSs are constructed. Based on the proposed interpretation together with exploitation of notion of Hausdorff distance, we have developed a novel way to calculate distance and similarity measures between q-ROFSs in the context of ET. Finally, proposed distance is utilized to develop OBP-GRA for the selection of COVID-19 vaccines. Relevant numerical simulations and applications to resolve complex daily life issues attest the reasonability, validity, practicality and suitability of interpretation of q-ROFSs in the frame work of ET.

### Future research directions

In the future, we will further investigate the possibility to extend our research work to hesitant fuzzy sets, Bipolar fuzzy sets, Neutrosophic fuzzy sets and aggregation operators with application to MCDM, pattern recognition, clustering, image processing etc.

## Data Availability

All the relevant data is included in the manuscript.
